# Antiviral drug discovery and development: challenges and future directions

**DOI:** 10.1038/s41392-025-02539-7

**Published:** 2026-02-25

**Authors:** Shaoqing Du, Xueping Hu, Ping Li, Shujing Xu, Meehyein Kim, Xinyong Liu, Peng Zhan

**Affiliations:** 1 Department of Medicinal Chemistry, Key Laboratory of Chemical Biology (Ministry of Education), School of Pharmaceutical Sciences, Cheeloo College of Medicine, Shandong University, Jinan, Shandong P. R. China; 2 College of Chemical Engineering, Qingdao University of Science and Technology, Qingdao, Shandong P. R. China; 3 Institute of Frontier Chemistry, School of Chemistry and Chemical Engineering, Shandong University, Qingdao, Shandong P. R. China; 4 Department of Medicinal Chemistry, School of Pharmacy, Qingdao University, Qingdao, China; 5 Infectious Diseases Therapeutic Research Center, Korea Research Institute of Chemical Technology (KRICT), Daejeon, Republic of Korea

**Keywords:** Medicinal chemistry, Drug screening

## Abstract

The coronavirus disease 2019 (COVID-19) pandemic has stimulated extensive endeavors toward the development of therapeutic interventions targeting severe acute respiratory syndrome coronavirus 2 (SARS-CoV-2) and human proteins for viral infection control, encompassing numerous potential drugs and thousands of patients participating in clinical trials. These concerted efforts have resulted in significant advancements in antiviral drug discovery and development. In this review, we present a comprehensive timeline detailing the development of antiviral drugs, tracing the progression from early viral inhibitors to modern broad-spectrum antiviral agents. We also outline the current status of advancements in antiviral drug discovery, encompassing target-based strategies, innovative mechanism-based approaches, and pharmacokinetic optimization. Furthermore, we discuss the challenges and future prospects gained from COVID-19 and other infectious diseases, covering knowledge of artificial intelligence strategies, the utilization of medicinal chemistry tools, and advancements in nanotechnology applications. The application of artificial intelligence in drug discovery is increasingly prevalent, particularly in the areas of protein structure prediction, drug target identification, and bioactivity forecasting. Nanotechnology has played a crucial role in the delivery of antiviral drugs and the development of vaccines, exemplified by the use of lipid nanoparticles in mRNA vaccines. Additionally, we highlight potential future directions for drug discovery, such as targeting membraneless organelles (liquid‒liquid phase separation).

## Introduction

When confronted with the threat of a viral outbreak, practical public health measures prioritize the use of vaccines and antiviral therapeutics.^1–3^ While vaccination initiatives exhibit promise in preventing infections, the critical role of antiviral drugs in effectively managing future human pathogens becomes evident.^4–7^ The appearance of SARS-CoV-2 variants with reduced vaccine efficacy strongly underscores the necessity for antiviral reserves.^8,9^ Consequently, this imperative need for antiviral agents propels researchers to employ innovative drug design strategies to discover novel therapeutic candidates.

There are two primary approaches to drug discovery: phenotypic drug discovery^10,11^ and target-based drug discovery (TBDD)^12–14^. Phenotypic drug discovery involves evaluating the impact of drug candidates on disease models for screening purposes. This approach has the advantages of expanding the “druggable” target space, demonstrating polypharmacology (targeting multiple targets), and identifying therapeutic agents with relatively low molecular weights.^15–17^ Current trends indicate that artificial intelligence (AI)-driven phenotypic screening is transitioning from empirical approaches to mechanism-oriented precision screening.^18^ This approach is particularly suitable when drugs with new mechanisms of action, especially first-in-class drugs, are needed or when the disease involves multiple biological pathways and may require multitarget interventions. Yu Rao et al. utilized a chemical library for phenotypic screening and discovered novel antiviral inhibitors. Through optimization from hit to lead, a new potent small molecule (RYL-634) was identified, which demonstrated excellent broad-spectrum inhibitory activity against various pathogenic viruses. The mechanism of action and potential targets of RYL-634 were further investigated via techniques such as active protein profiling analysis. Ultimately, it was confirmed that human dihydroorotate dehydrogenase (DHODH) is the primary target of RYL-634.^19^ With respect to this approach, more sophisticated phenotypic models that accurately reflect the pathophysiology of complex diseases are needed.

TBDD focuses on specific targets closely associated with the disease mechanism.^20^ With the advent of the molecular biology revolution and the human genome era, precision medicine emphasizes detailed molecular mechanisms, making TBDD the prevailing direction in traditional pharmaceutical research.^21,22^ Concurrently, target-oriented methodologies are evolving toward system-level regulation through the application of multiomics technologies. This is exemplified by the identification of TRAF2-interacting kinase and NCK-interacting kinase as pivotal targets for pulmonary fibrosis through the analysis of multiomics data via AI. Using AI-driven methodology, candidate molecules were designed and subsequently screened and optimized, leading to the discovery of INS018_055 (IC_50_ = 27.14 nM).^23^ The compound is currently undergoing phase II clinical trials. Similarly, TBDD faces several challenges, including concerns regarding the long-term use of direct-acting antiviral drugs, which may lead to the emergence of drug-resistant variants in patients.^24^

The methods of phenotype screening and TBDD differ fundamentally in terms of their scientific logic, technical pathways, and application scenarios. Phenotype screening directly targets disease phenotypes (such as viral replication inhibition) to assess drug activity, whereas TBDD first identifies molecular targets associated with the disease (such as key enzymes) before designing compounds to modulate their functions. Consequently, phenotype screening has advantages in the early stages of drug discovery, particularly for diseases where the mechanisms are not well understood or where effective therapeutic options are lacking. Additionally, it plays a significant role in the identification of novel first-in-class drugs. In contrast, TBDD is more advantageous during the later stages of drug development. This approach is especially efficient when optimizing drugs for known targets and developing follow-up medications (follower drugs), allowing for more effective screening and design processes. The future of drug discovery will profoundly integrate these two strategies, facilitating a comprehensive breakthrough in the journey from phenomenon to essence.

Despite significant advancements in drug screening and AI, the discovery of antiviral drugs faces several challenges. These include a high mutation rate of viruses, the propensity for developing resistance, a wide variety of viral types with few commonalities, and difficulties in balancing drug efficacy with safety during the research and development process. Additionally, the complexity of the clinical trial design and patient selection further complicates these efforts. The high costs associated with research and development, coupled with substantial risks and limitations related to the application of new technologies—such as machine learning (ML), which requires extensive data support—pose additional hurdles. The accumulation of large datasets in the field of antiviral drug development remains insufficient. On October 19, 2021, Atea Pharmaceuticals announced that its investigational oral COVID-19 medication AT-527 did not meet the primary endpoint in the global phase II study. Although AT-527 exhibited promising antiviral activity in vitro and in animal models, it has not achieved the expected efficacy in human clinical trials. This outcome highlights the substantial challenges encountered during the translation from laboratory research to clinical application. Factors such as the pharmacokinetic properties of the drug within humans and complex interactions between the drug, virus, and host cells may influence both its actual efficacy and safety profile. To address these multifaceted limitations and challenges, researchers, enterprises, and relevant institutions must collaborate closely. The continuous exploration of innovative strategies and methodologies is crucial for increasing both the success rate and the efficiency of antiviral drug development.

**Fig. 1:**
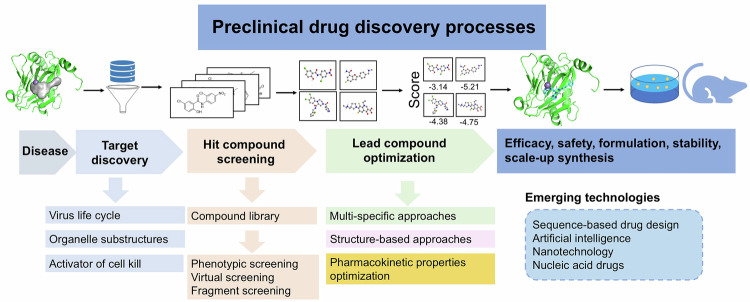
Preclinical drug discovery processes, strategies, and techniques. The illustration depicts a process for TBDD. The content includes identifying targets based on diseases, screening potential candidates, optimizing lead compounds, and addressing drug-like properties. The incorporation of emerging technologies, including sequence-based drug design, artificial intelligence, nanotechnology, and nucleic acid therapeutics, has markedly expedited the drug discovery process

Since the onset of 2020, the emergence of the novel coronavirus has prompted pharmaceutical researchers worldwide to actively pursue antiviral drug development.^25–28^ The convergence of efforts from the biotechnology and pharmaceutical industries, academia, and diverse fields of expertise remains pivotal for successful advancements in medicinal innovation.^29–31^ The globally dedicated coronavirus disease Moonshot project, led by Frank von Delft et al., has advanced from the fragment screening of molecules binding to the SARS-CoV-2 main protease (M^pro^) to the identification of the clinical candidate DNDI-6510 in just 18 months.^32,33^ As of March 2024, seven oral drugs for COVID-19 have been launched in China, including azvudine, nirmatrelvir, molnupiravir, simnotrelvir, deuremidevir hydrobromide, leritrelvir, and atilotrelvir. (https://www.nmpa.gov.cn/datasearch/home-index.html#category=yp) Throughout this process, numerous groundbreaking strategies and methodologies have surfaced, akin to a gathering of eminent scholars across research borders. The COVID-19 pandemic has not only emerged as a global health crisis but also marked a pivotal moment in the field of antiviral drug development. This review systematically summarizes the scientific breakthroughs and technological innovations spurred by the pandemic, laying a solid foundation for strategic planning in antiviral drug development in the post-COVID-19 era (Fig. 1).

## The development of antiviral drugs and basic principles of drug discovery

### Development of antiviral drugs

#### Approval of idoxuridine (the first approved antiviral drug)

**Fig. 2:**
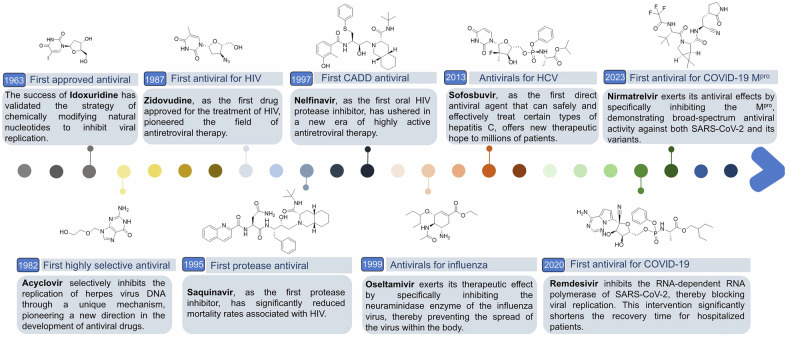
The timeline of milestones for the development of antiviral drugs. In the late 1970s, acyclovir was discovered to inhibit the DNA replication of herpes simplex virus (HSV) at concentrations significantly lower than those affecting cellular DNA synthesis, marking the beginning of a new era in antiviral chemotherapy. In the 1980s, in response to the rampant spread of HIV/AIDS, zidovudine emerged as the first clinically approved antiretroviral drug. Zanamivir was introduced in 1993, followed by oseltamivir in 1997; both became early antiviral medications notable for their oral administration and high bioavailability. Since entering the 21st century, there has been rapid development of antiviral drugs, with several becoming available for combating COVID-19

In 1963, idoxuridine (Fig. 2) emerged as the first antiviral drug.^34^ This pyrimidine antiviral agent competitively inhibits thymidine kinase and DNA polymerase, thereby hindering viral replication. Its success validated the strategy of chemically modifying natural nucleotides to disrupt viral replication, directly inspiring the development of subsequent nucleoside-based drugs such as acyclovir and zidovudine. It is primarily indicated for the treatment of herpetic keratoconjunctivitis in humans; however, its use is limited to topical applications because of its inability to differentiate between viral and host cellular functions.

During this period, antiviral drugs such as moroxydine,^35,36^ ancitabine,^37,38^ vidarabine,^39^ and cytarabine^40,41^ emerged. These compounds have some efficacy in inhibiting viral replication and treating associated diseases; however, the variety of available antiviral drugs remains limited.

#### The emergence of acyclovir: the first selective, virus-activated prodrug

Acyclovir revolutionized antiviral therapy as the first selective, virus-activated prodrug. Its mechanism relies on viral thymidine kinase to initiate phosphorylation, converting it into a monophosphate form, which is further phosphorylated by host kinases to its active triphosphate form. The active triphosphate form of acyclovir selectively inhibits herpesvirus DNA polymerase, effectively blocking viral DNA synthesis.^42^ It is the preferred treatment for herpesvirus infections. Acyclovir is efficacious in managing various conditions associated with herpesvirus infections, including herpes keratitis, herpes simplex, and herpes zoster, with minimal adverse effects. Both its oral and intravenous formulations are extensively utilized in clinical settings. The success of acyclovir inspired prodrug optimization (e.g., valacyclovir for improved bioavailability) and antiviral strategies emphasizing enzyme-specific activation.

#### Approval of zidovudine: the first antiretroviral drug approved for the treatment of acquired immune deficiency syndrome (AIDS)

With the discovery and epidemic of AIDS, the need for anti-human immunodeficiency virus (HIV) drugs has become urgent. In 1987, zidovudine was approved for the treatment of AIDS.^43^ By inhibiting nucleotide reverse transcriptase (RT), it blocks viral reverse transcription, replication, and translation processes, suggesting the potential for the treatment of AIDS. Since then, a variety of anti-HIV drugs have emerged (Table 1).

**Table 1:** Therapeutic targets and clinical advancements in the development of small molecule antiviral drugs (FDA-approved drugs and ongoing clinical trials)

Virus	Mechanisms of action	Drug name	Time-to-market or clinical trials
HIV	Nucleoside RT inhibitors	Zidovudine	1987
		Didanosine	1991
		Zalcitabinea	1992
		Stavudine	1994
		Lamivudine	1995
		Abacavir	1998
		Emtricitabine	2003
		Tenofovir	2008
		Islatravir	Phase III
	Nonnucleoside RT inhibitors (NNRTIs)	Nevirapine	1996
		Delavirdine	1997
		Efavirenz	1998
		Etravirine	2008
		Rilpivirine	2011
		Doravirine	2018
		GS-5894	Phase I
	Integrase inhibitors	Raltegravir	2007
		Elvitegravir	2012
		Dolutegravir	2013
		Bictegravir	2018
		Cabotegravir	2020
		GS-1720	Phase II/III
		VH4524184	Phase II
		GS-6212	Phase I
	gp120 inhibitor	Forstemsavir Tromethamine	2020
	CCR5 coreceptor inhibitor	Maraviroc	2007
	Capsid (CA) inhibitor	Lenacapavir	2022
	Protease inhibitors	Saquinavir	1995
		Indinavir	1996
		Ritonavir	1996
		Nelfinavir	1997
		Amprenavira	1999
		Lopinavir	2000
		Fosamprenavir	2003
		Atazanavir	2003
		Tipranavir	2005
		Darunavir	2006
	Attachment Inhibitor	Fostemsavir	2020
	Entry inhibitors	Enfuvirtide	2003
		Albuvirtide	Phase III
HBV	RT inhibitors	Lamivudine	1991
		Tenofovir disoproxil fumarate	2001
		Adefovir dipivixil	2002
		Entecavir	2005
		Telbivudine	2006
		Tenofovir alafenamide	2016
	Entry inhibitors	Hepcludex	Phase II
		Hepalatide	Phase II
	CA inhibitors	Morphothiadin	Phase III
		QL-007	Phase II
Influenza virus	M2 proton channel antagonists	Rimantadine hydrochloride	1993
		Amantadine hydrochloride	1987
	NA inhibitors	Zanamivir	1999
		Oseltamivir	1999
		Peramivir	2014
	Cap-dependent endonuclease inhibitor	Baloxavir	2018
SARA-CoV-2	RNA-dependent RNA polymerase (RdRp) inhibitors	Remdesivir	2020
		Molnupiravir	2021
	M^pro^ inhibitors	Nirmatrelvir	2023
		WPV01	Phase III
HCV	RNA polymerase inhibitor	Ribavirin	1985
	NS3/4 A protease inhibitors	Telaprevir	2011
		Boceprevir	2011
		Simeprevir	2013
		Paritaprevir	2014
		Grazoprevir	2016
		Voxilaprevir	2017
		Glecaprevir	2017
	NS5A inhibitors	Ledipasvir	2014
		Ombitasvir	2014
		Daclatasvir	2015
		Velpatasvir	2016
		Elbasvir	2016
		Pibrentasvir	2017
		Ruzasvir	Phase III
		Odalasvir	Phase II
	NS5B polymerase inhibitors	Sofosbuvir	2013
		Dasabuvir sodium	2014
		Bemnifosbuvir	Phase III
		Adafosbuvir	Phase II
Herpes simplex virus (HSV)	DNA polymerase inhibitors	Idoxuridine	1963
		Cytarabine	1969
		Vidarabine	1976
		Acyclovir	1982
		Famciclovir	1994
		Valacyclovir	1995
		Penciclovir	1996
	Thymidylate synthase inhibitor	Trifluridine	1982
	Entry inhibitor	Docosanol	2000
Cytomegalovirus (CMV)	DNA-terminal enzyme inhibitor	Letermovir	2017
	DNA polymerase inhibitors	Foscarnet	1991
		Cidofovir	1996
		Valganciclovir	2001
		Ganciclovir	2003
		Maribavir	2021
	Antisense Oligonucleotides	Fomivirsen	1998
Human papillomavirus (HPV)-related diseases	Stimulates cytokine production	Imiquimod	1997
	Immunomodulator	Sinecatechins	2006
	Interrupts cell division	Podofilox	1990

In the 1990s, valaciclovir,^44^ famciclovir,^45^ and penciclovir,^46–48^ among other drugs, were successfully developed. Once these drugs are converted into triphosphate compounds within the body, they effectively interfere with viral DNA polymerase, thereby inhibiting viral DNA replication. They are particularly efficacious against herpes DNA viruses, including herpes simplex virus types I and II and varicella-zoster virus. This development significantly expanded the range of antiviral medications available for treating herpes viruses. Additionally, during this period, a variety of antiviral drugs targeting different viruses emerged, such as the combination of interferon (IFN) and ribavirin for the treatment of hepatitis C virus (HCV), providing effective therapeutic options for a broader spectrum of viral diseases.

#### Computer-aided drug design (CADD) facilitates drug discovery

CADD has played a crucial role in the development of antiviral drugs, such as nelfinavir,^49–51^ which was identified in the 1990s for the treatment of HIV infection. However, the process was relatively inefficient at the time because of inaccurate calculations and limited computational power, allowing only the docking of approximately 100 compounds at once. Additionally, both the target and the drug were required to remain rigid in a lock-and-key fashion during the docking process. This rigid docking is uncommon in reality because proteins undergo thermally driven internal motions that cause the binding site shape to fluctuate.^52^

The development and application of neuraminidase (NA) inhibitors, such as oseltamivir^53–55^ and zanamivir,^56–58^ have provided potent tools for the treatment and prevention of influenza, particularly in managing influenza pandemics. The rational computer-aided design of zanamivir heralded a new era in the development of antiviral drugs.

Since the beginning of the 21st century, antiviral drugs have entered an advanced stage of development, leading to the development of various novel anti-HIV drugs, including protease inhibitors^59–61^ and integrase inhibitors^62–64^. When combined with traditional RT^65,66^ inhibitors, these drugs form a highly effective antiretroviral therapy regimen, significantly enhancing the treatment outcomes for AIDS patients and extending patient lifespans while improving their quality of life.

The computational power of supercomputers has increased approximately one millionfold since the 1990s. Consequently, rigid docking of over a billion compounds can now be accomplished within days. Virtual high-throughput screening thus outperforms its experimental counterpart, enabling the rapid identification of highly potent compounds. Moreover, molecular dynamics simulations facilitate the calculation of internal protein movements, allowing for the screening of drug candidates via ensemble docking,^67,68^ which considers the various conformations of binding sites. This method is more realistic than rigid docking and has proven effective, particularly in HIV drug discovery efforts since the early 2000s. Jeremy C. Smith’s laboratory has successfully identified experimentally validated hits against all 16 proposed protein targets over the past few years via ensemble docking.^52^

#### Viral biology contributes to drug discovery

With in-depth investigations into the biological characteristics and pathogenesis of viruses, an increasing number of novel antiviral drug targets have emerged. For example, protease inhibitors,^69,70^ polymerase inhibitors^71,72^ targeting HCV (sofosbuvir)^73–76^ and fusion inhibitors targeting respiratory syncytial virus^77,78^ have been identified, offering clear directions for the development of more specific and efficacious antiviral therapies.

The advent of nucleoside analogs, including lamivudine, adefovir dipivoxil,^79–81^ and entecavir,^82–86^ has provided a more efficacious alternative for the management of chronic hepatitis B. By inhibiting hepatitis B virus (HBV) replication, these medications effectively retard disease progression and diminish the likelihood of developing cirrhosis and hepatocellular carcinoma.

#### The emergence of new technologies has opened a new path for drug discovery

Proteolysis-targeting chimera (PROTAC) molecules targeting specific viral proteins are designed to facilitate their specific binding and recruitment of E3 ubiquitin ligases, thereby attaching ubiquitin tags to these viral proteins. This tagging enables recognition and degradation by the proteasome, effectively blocking viral replication and transmission. Haibing Zhou and colleagues developed a series of PROTAC compounds based on the oseltamivir scaffold. Notably, the PROTAC compound significantly degrades the influenza virus NA, effectively inhibits H1N1 influenza virus replication, and has potent activity against oseltamivir-resistant strains. This work offers a novel approach for the development of anti-influenza drugs.^87^

#### Accelerated discovery of antiviral drugs during the COVID-19 pandemic

Following the outbreak of the novel coronavirus, researchers worldwide swiftly initiated research and development of antiviral drugs, achieving significant progress within a short timeframe. Notably, drugs such as remdesivir^88–92^ and nirmatrelvir^93,94^ have provided crucial support in combating the COVID-19 pandemic. In an extended phase of an open-label study and a retrospective real-world investigation, a total of 1974 patients receiving remdesivir in conjunction with standard treatment were included, alongside 1426 patients receiving standard treatment alone. The results indicated that remdesivir significantly reduced the risk of mortality and markedly increased the likelihood of patient discharge at 28 days.^95^

Since the approval of the first antiviral drug, idoxuridine, by the Food and Drug Administration (FDA), over 100 antiviral drugs have been subsequently approved by the FDA.^25,96^ We summarize the therapeutic targets and clinical progress of several antiviral drugs (Table 1). With the development of technology and the crossover of disciplines, more small-molecule drugs will be discovered.

### Basic principles of drug discovery

In the evolution of modern medicine, numerous therapeutic strategies have advanced significantly; however, additional treatment alternatives remain essential for the prevention and management of both prevalent and rare diseases.^97^ The process of drug development is protracted, fraught with risks, and financially demanding. Approximately 90% of drugs that enter clinical trials fail to gain approval, primarily owing to inadequate efficacy.^98,99^ On the basis of an analysis of data between the years 2009 and 2018, it was estimated that the median investment in research and development required to bring a new drug to market amounts to $1,142 million,^100^ yet the low success rates translate into substantial social costs amounting to tens of billions of dollars annually.

To increase the success rate of drug discovery, contemporary pharmaceutical research is undergoing a critical transformation from follow-on generics to original innovations, necessitating multidimensional strategies to overcome traditional bottlenecks. In the realm of target and mechanism innovation, multitarget collaborative drug design transcends the limitations of conventional single-target approaches.^101^ Amidst technological advancements, the deep integration of AI with experimental methods enables ML to optimize molecular generation, toxicity prediction, and multiobjective optimization, thereby shortening preclinical development cycles.^102^ Furthermore, interdisciplinary collaborative innovation enables the exploration of modern pharmacological mechanisms of active components in traditional Chinese medicine (such as artemisinin and arsenic trioxide).^103,104^ By validating these mechanisms against medicinal targets, novel drugs can be developed.

Failures in drug development can often be attributed to gaps in our understanding of the biological underpinnings of human diseases, an overreliance on nonhuman models, and deficiencies such as low specificity, suboptimal pharmacological properties, or severe adverse reactions to drugs.^105^ Recent advancements in human genome sequencing and high-throughput sequencing technologies have deepened our comprehension of genetic aberrations in cancer and the molecular mechanisms underlying over 4000 rare, monogenic disorders.^106,107^ Moreover, these technological advancements have facilitated our understanding of the associations between thousands of genetic variants and complex, polygenic common diseases.^108–110^ Building on these technological advancements, a deeper understanding of the principles of drug discovery will expedite the process of identifying new therapeutic agents.

Despite the continuous evolution of drug discovery strategies, contemporary pharmaceutical research and development faces multiple challenges across various dimensions, including technology, economics, policy, and ethics. First, investment in research and development has been steadily increasing; however, social capital lacks confidence in long-term returns—especially following a cooling period in global pharmaceutical investments after 2022. Second, even with rigorously selected candidate drugs, the failure rate in phase III clinical trials remains alarmingly high. The difficulties associated with annotating real-world data, coupled with patient privacy protections that restrict data sharing, adversely affect both AI model training and clinical prediction accuracy. Furthermore, high-quality experimental data are scarce and costly to obtain; publicly available datasets often lead to positive results, which can skew model training outcomes. Additionally, molecules designed by generative AI may be either nonsynthesized or possess unknown toxicity profiles; these models also suffer from poor interpretability and insufficient multiobjective optimization capabilities. In light of these challenges, it is essential for stakeholders to collaborate effectively—not only to address technological bottlenecks but also to establish a supportive policy and market environment conducive to innovation. Ultimately, this collaborative effort aims to facilitate a transformation from imitation to leading innovation.

#### Target identification and validation

Drug targets are biological macromolecules that exhibit pharmacodynamic functions within the body and can be modulated by drugs, such as specific proteins and nucleic acids.^111^ The identification of these targets involves a comprehensive understanding and analysis of the disease mechanism, which is achieved by comparing genomic and proteomic differences between normal and diseased tissues to pinpoint key proteins.^112–114^

The general process of target identification and validation involves searching for disease-related biomolecular markers and conducting functional studies on these biomolecules to identify potential targets for drug candidates. Target validation is designed to confirm a direct connection between the target and the therapeutic area, demonstrating that modulating the target could yield therapeutic benefits for the disease. This validation encompasses a range of methods, from in vitro experiments to in vivo studies using whole-animal models that mimic patient conditions, analyzing the impact on gene and protein expression, and ultimately validating target modulation in clinical settings.^112,115^

Genomics, proteomics, biochip technology, and other high-throughput methods are employed to acquire information on the expression, modification, interaction, and other characteristics of biomolecules under both diseased and normal conditions.^116–118^ Through bioinformatics analysis, biological molecules that exhibit abnormal expression or dysfunction in diseases or those that are closely associated with the onset and progression of diseases are identified as potential drug targets.^119^

A promising drug target should exhibit a well-defined causal relationship with the onset, progression, or pathophysiological processes of the disease, and its modulation should effectively alleviate symptoms or slow disease progression.^120,121^ Additionally, the druggability of the target must be considered, meaning that small-molecule compounds, antibodies, or other bioactive molecules can be identified to bind specifically and modulate its activity.^111^

Targets can be validated via gene, protein, and animal models. Gene knockout, gene silencing, and other technologies, such as the CRISPR/Cas9 system or RNA interference, can be employed in cells or animal models to specifically knock out or reduce the expression of the target gene.^122,123^ By observing the impact on the disease phenotype, researchers can establish the causal relationship between the target gene and the disease.^124^

By developing specific antibodies or small molecule inhibitors against target proteins, functional experiments can be conducted in both cellular and animal models. These experiments include assessing the activity, intracellular localization, and interactions with other proteins of the target protein, as well as evaluating the amelioration of disease-related phenotypes.^23,125–128^ This approach aims to validate the function of the target protein and its potential as a viable drug target.

The development of cell and animal models pertinent to the disease allows for the verification of the efficacy of the target and the impact of drug interventions.^129^ In the cell model, the main evaluation is drug efficacy and safety. In animal models, the evaluation focuses on improvements in disease symptoms, pathophysiological markers, and survival rates.^130^ In animal models, different species exhibit varying levels of susceptibility to the same virus. For example, HBV exhibits highly restricted species tropism, infecting only a limited number of species, including humans, chimpanzees, and treeshrews.^131^ Additionally, the ability of the innate immune systems of different species to recognize and respond to viral infections varies. Pattern recognition receptors, including toll-like receptors (TLRs), may display species-specific differences in expression levels and functional activity, which in turn influences the efficiency of virus detection and downstream signaling pathways.^132^ The differences in immune responses and incomplete similarities in disease manifestations due to species differences render animal models somewhat limited. These species-specific differences hold significant implications for virology research and drug development. When selecting an animal model, it is crucial to consider its relevance to human disease to ensure the accuracy and extrapolability of the study findings.

PrecisionLife’s combinatorial analysis platform is utilized to analyze patient datasets to identify novel therapeutic targets and patient-specific stratified biomarkers for central nervous system diseases, thereby providing a robust foundation for target selection.^133,134^

#### Antiviral drug targets

The virus‒host interaction network encompasses three primary antiviral drug targets: viral targets, host targets, and viral antagonism to host innate immunity. Among these, viral targets are traditionally considered the primary focus, whereas host targets are given secondary importance.^135–137^ Viral antagonism to host innate immunity has often been overlooked during the development of antiviral drugs.^138^

**Fig. 3:**
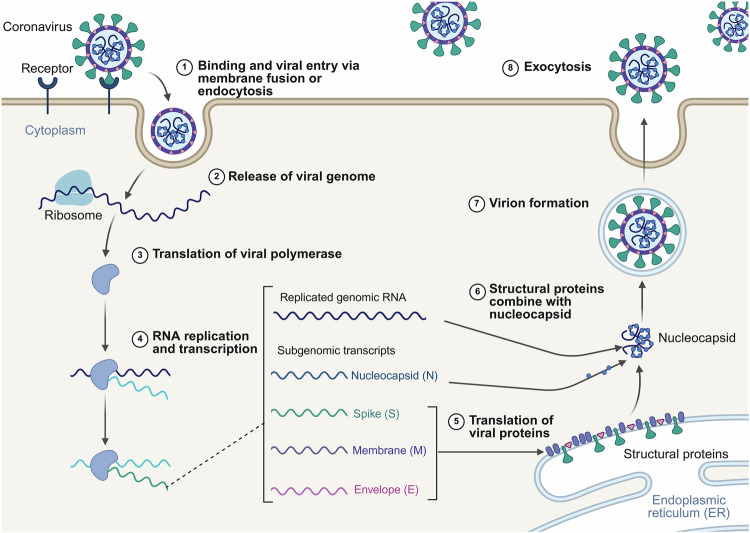
The life cycle of SARS-CoV-2. The following are reported inhibitors that target various stages of the viral life cycle: spike (S) inhibitors, papain-like protease (PL^pro^) inhibitors, M^pro^ inhibitors, nonstructural protein 12 (NSP12) inhibitors, NSP13 inhibitors, NSP14 inhibitors, NSP15 inhibitors, NSP16 inhibitors, and nucleocapsid (N) protein inhibitors

Different stages of the viral life cycle, including adsorption, penetration, uncoating, replication, gene expression, assembly, and release, serve as potential targets for antiviral intervention (Fig. 3).^139,140^ During the development of antiviral drugs, modulating the viral life cycle constitutes the primary mechanism of action for most antiviral agents.^25^

Host-targeting antiviral drugs represent a therapeutic strategy that curtails viral replication by interfering with specific host cell factors. Host cell kinases are pivotal in the replication processes of numerous viruses, making them promising targets for antiviral drug development.^141–143^ Both Dengue virus and Zika virus exploit the receptor tyrosine kinase AXL as an entry mediator for host infection, whereas the epidermal growth factor receptor functions as a coreceptor for adeno-associated virus serotype 6 and human cytomegalovirus to facilitate their entry into the host.^144–146^ Moreover, adaptor-associated kinase 1 (AAK1) governs the intracellular trafficking of various viruses, suggesting that AAK1-targeted drugs could serve as optimal candidates for the creation of broad-spectrum antiviral agents.^147^ DHODH, which is located in the inner mitochondrial membrane, is a critical enzyme responsible for catalyzing the de novo synthesis pathway of pyrimidine nucleotides. Since viral replication necessitates pyrimidine nucleotides derived from de novo synthesis to fulfill its nucleic acid demands, inhibiting DHODH activity can effectively impede the biosynthesis of viral DNA and RNA, thereby suppressing viral replication. Ke Xu et al. identified a series of host-targeted DHODH inhibitors capable of exhibiting broad-spectrum antiviral efficacy by inhibiting viral genome replication and modulating immune responses.^148^

The interaction between viruses and hosts is inherently a dynamic process. Upon recognition of influenza A virus (IAV) and SARS-CoV-2 RNA by the host’s pattern recognition receptors, the antiviral innate immune signaling pathway is promptly activated, leading to the induction of host restriction factors such as IFN-stimulated genes (ISGs), which in turn inhibit one or multiple stages of the viral replication cycle.^149^ Concurrently, viruses have developed diverse regulatory mechanisms at the transcriptional, translational, posttranslational, and epigenetic inheritance levels to counteract host innate immunity, thereby facilitating their effective replication through command of the host cell’s translation machinery and increasing the expression of host factors. Notably, this interplay between host innate immunity and viral evasion strategies constitutes a complex virus‒host interaction network.^150^ Leveraging the virus‒host interaction network as an antiviral drug target, particularly for IAV and SARS‒CoV‒2, offers a promising approach to address the challenge of viral drug resistance.^138^

#### Signaling pathways for antiviral agents

**Fig. 4:**
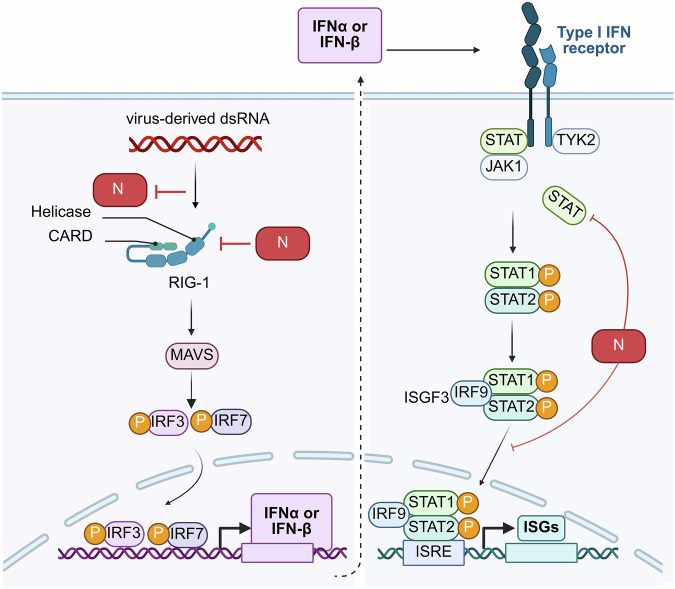
The host cell’s innate immune processes involve the SARS-CoV-2 N protein. N proteins interact with RIG-I and inhibit RIG-mediated production of IFN-β. These N proteins engage with RIG-I through the helicase domain of DExD/H-box helicases, which possess ATPase activity and play crucial roles in binding immunostimulatory RNAs. Consequently, N proteins attenuate the IFN-β response by targeting the initial step of interferon activation. N proteins antagonize type I IFN signaling by inhibiting the phosphorylation and nuclear translocation of signal transducer and activator of transcription 1 and 2 (STAT1 and STAT2). The binding of secreted type I IFN to its receptors on adjacent cells can initiate the phosphorylation of preassociated Janus kinase 1 (JAK1) and tyrosine kinase 2 (TYK2), which subsequently phosphorylate the receptors. This process leads to the recruitment and phosphorylation of STAT1 and STAT2. In cells infected with SARS-CoV-2, N proteins can disrupt the interactions between STAT1 and JAK1 as well as between STAT2 and TYK2 by competitively binding to both STAT1 and STAT2, thereby inhibiting their phosphorylation. Consequently, N proteins further diminish the subsequent nuclear translocation of the interferon-stimulated gene factor 3 (ISGF3) transcription complex, ultimately suppressing the expression of ISGs.^154^

Antiviral therapy can specifically modulate signaling pathways through various mechanisms to enhance the host antiviral response or inhibit viral replication and transmission (Fig. 4). The antiviral signaling pathways primarily include the IFN signaling pathway, the retinoic acid inducible gene I (RIG-I) signaling pathway, the cyclic GMP–AMP synthase-stimulator of IFN genes (cGAS-STING) signaling pathway, and the TLR signaling pathway, among others.^151–153^

Protein‒protein interactions are essential for cellular signaling and transduction, making them highly attractive targets for therapeutic drug development. Once deemed undruggable, these targets have become increasingly viable owing to significant advancements in technology and research over the past two decades.^154^

The IFN signaling pathway is one of the critical mechanisms in antiviral immune responses. It confers resistance to viral infections by inducing an antiviral state within cells. Upon viral infection, IFN secreted by cells binds to its receptor, activating JAK. Subsequently, JAK phosphorylates STAT (Fig. 4), leading to dimer formation and nuclear translocation, which initiates the expression of a series of ISGs and exerts antiviral effects.^151,155–157^ IFN-*β* can activate the JAK-STAT pathway, promoting the expression of numerous proinflammatory cytokines, including tumor necrosis factor-alpha (TNF-*α*) and interleukin-6 (IL-6), thereby initiating the antiviral immune response in the host.^158,159^ The SARS-CoV-2 N protein disrupts the interactions between STAT1 and JAK1, as well as between STAT2 and TYK2, by competitively binding to both STAT1 and STAT2. This interference subsequently inhibits their phosphorylation and prevents their nuclear translocation in 293 T cells.^160^

The nuclear factor kappa-B (NF-*κ*B) pathway can be activated during the early stages of viral infection. On the one hand, this activation promotes the production of inflammatory factors, triggering an inflammatory response to combat the virus. On the other hand, the activation of NF-*κ*B can also increase IFN transcription, thereby strengthening the antiviral immune response.^161,162^ Additionally, certain ISG products can regulate the NF-*κ*B pathway, contributing to a complex regulatory network that collectively maintains the antiviral state of the organism.^163^ After binding to viral RNA, the SARS-CoV-2 N protein undergoes robust liquid–liquid phase separation (LLPS), which facilitates the recruitment of TAK1 and the IKK complex—key kinases in NF-*κ*B signaling—thereby increasing NF-*κ*B activation.^164^

Viral infections can activate inflammasome signaling pathways, notably the nucleotide-binding oligomerization domain, leucine-rich repeat and pyrin domain-containing 3‌ inflammasome, which leads to the maturation and secretion of the proinflammatory cytokines IL-1*β* and IL-18, thereby eliciting an inflammatory response.^165–168^ This inflammation can, in turn, modulate the activity of the IFN signaling pathway. Excessive inflammation has the potential to suppress the antiviral immune response, cause tissue damage, and impair the body’s antiviral efficacy. The Zika virus exploits the crosstalk between the IFN and inflammasome pathways to induce hyperactivation of the inflammasome, culminating in immunopathological damage.^169^

RIG-I (Fig. 4a) plays a crucial role in the antiviral innate immune response by recognizing viral RNA and transmitting signals to two key pathways: the IFN-*β* signaling pathway and the NF-*κ*B signaling pathway.^170–173^ Its downstream effector, mitochondrial antiviral signaling protein, functions as a critical signal transducer that activates kinases such as TANK binding kinase 1 and inhibitor of *κ*B kinase, subsequently leading to the activation of transcription factors such as IFN regulatory factor 3 and NF-*κ*B, thereby initiating the antiviral immune response.^174^ SARS-CoV-2 ORF6, ORF8, and N function as potent antagonists of IFN.^160^

The cGAS-STING signaling pathway primarily involves viral DNA within the cytoplasm and, upon activation, triggers the production of type I IFNs and other inflammatory mediators, thereby initiating an antiviral immune response. Moreover, there is a significant interaction and synergistic effect between the cGAS-STING and RIG-I signaling pathways, which collectively enhance the body’s antiviral immune response.^175–177^ β-Arrestin 2 can directly interact with cGAS, promoting the binding of cGAS to DNA and the synthesis of cyclic GMP-AMP (cGAMP), thus positively modulating the type I IFN pathway and exerting an anti-infective role.^158,178^ SARS-CoV-2 infection results in the accumulation of released mitochondrial DNA, which subsequently activates cGAS, leading to the initiation of IFN-I signaling. The N protein of SARS-CoV-2 inhibits the DNA recognition capacity of cGAS, thereby impairing cGAS-induced IFN-I signaling.^179^

The utilization of drugs to modulate the host immune system and augment its ability to identify and eliminate viruses is a promising approach.^180–182^ PAV-104 effectively reverses the induction of the IFN-I response by SARS-CoV-2, as well as the maturation of the nucleoprotein signaling pathway that is known to facilitate coronavirus replication. It represents a promising therapeutic candidate for COVID-19 and is characterized by a mechanism of action that is distinct from current clinical management strategies.^183^ Certain immunomodulators can increase the activity of immune cells and increase antibody production, thereby assisting the body in more effectively combating viral infections.^184^

In addition to the general signaling pathways affected during viral infections mentioned above, TLRs currently hold significant potential in the development of antiviral drugs. TLRs are a class of pattern recognition receptors that can identify viral nucleic acids and other pathogen-associated molecular patterns, thereby activating antiviral immune responses, and serve as the primary barrier that protects the organism from pathogenic invasion.^185^

TLR3 is a pattern recognition receptor located in endosomes that recognizes double-stranded RNA from viruses and parasites. Upon activation, TLR3 secretes IFN-α/β, establishing a local antiviral state that restricts viral replication at the site of infection.^186,187^

TLR7 is expressed primarily in intracellular vesicles and is capable of recognizing exogenous single-stranded RNA viruses.^188^ TLR7 agonists, such as imiquimod, have been approved for the treatment of warts caused by HPV. Imiquimod activates TLR7, inducing the production of type I interferons and enhancing antiviral responses. GS-9620 is an oral selective TLR7 agonist. It exerts its antiviral effects by activating the TLR7 signaling pathway, which induces the production of type I interferons and other antiviral factors. In chimpanzees infected with HBV, this compound effectively reduces the viral load in both plasma and liver tissues.^189^

Certain host cell proteins are crucial for viral infection and replication, making them viable targets for antiviral drugs.^190–192^ In vitro studies indicate that viral infection induces the degradation of β-arrestin 2, thereby promoting immune evasion. Conversely, the β-blocker carvedilol has been demonstrated to restore β-arrestin 2 expression, which is essential for maintaining the antiviral immune response. These findings suggest that carvedilol may be repurposed as a potential candidate for antiviral drug development.^158^

#### Drug screening

Drug screening can be categorized into two primary approaches: target-based screening and phenotypic screening. TBDD relies on an in-depth understanding of the structure and function of disease-related biological targets, such as proteins and nucleic acids, to design drug molecules that can specifically interact with these targets and modulate their activities, thereby achieving therapeutic effects.^193^

**Fig. 5:**
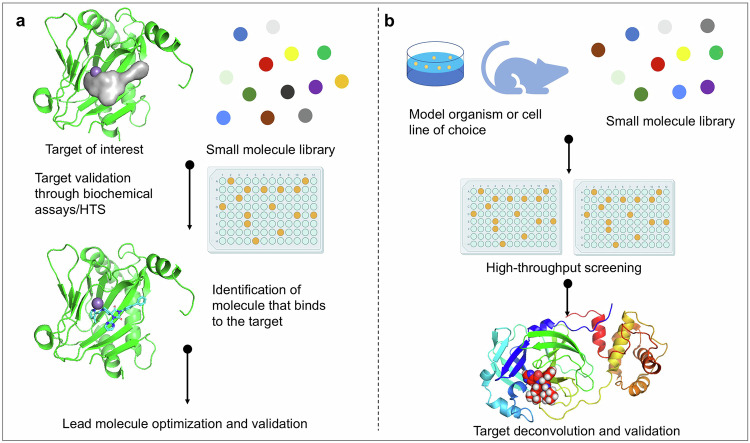
Fundamental processes of target-based screening (**a**) and phenotypic screening (**b**). The target-based screening focuses on specific biological macromolecules. This approach allows for the early clarification of a drug’s molecular mechanism of action and facilitates in-depth investigations into the interactions between drugs and specific targets through techniques like mutation analysis and crystallography. Phenotype-based screening is a strategy that identifies molecules exhibiting specific biological effects within cellular or animal models. This method directly observes the impact of compounds on cell or organism phenotypes. Such an approach enables screening in environments that more closely resemble physiological conditions, allowing for the discovery of new targets and mechanisms of action that may be difficult to identify through target-based screening

When the three-dimensional structure of the target protein is known, CADD technology can be employed to perform virtual screening across extensive compound databases (Fig. 5a).^194–197^ This process leverages the specific characteristics of the target protein’s binding site and the interaction modes between the site and small molecular compounds, including hydrogen bonding, van der Waals forces, and electrostatic interactions. Compounds that exhibit a rational binding pattern and high predicted affinity for the target protein are then selected as lead compounds.^198^

If a small-molecule ligand is known to be active against the target, one can search for chemically similar structures in compound databases via methods such as shape similarity,^199^ pharmacophore modeling,^200^ or ML,^201^ and screen for potential hit compounds with similar activities.^196,202–205^

The Schrödinger software platform offers a comprehensive suite of tools and methodologies for structure-based drug design, encompassing protein structure prediction and modeling, drug molecular docking, molecular dynamics simulations, and beyond. This software is extensively utilized in the pharmaceutical industry to assist researchers in rapidly and accurately predicting drug–molecule–target interactions, thereby expediting drug discovery and optimization processes.^206–208^

The DrugFlow drug discovery platform harnesses AI and ML technologies in conjunction with protein structure data to virtually screen and optimize a vast array of compounds, thereby accelerating the identification and assessment of potential drug candidates. Additionally, the platform is capable of predicting pharmacokinetic profiles, toxicity, and other critical drug properties, which enhances the overall success rate of drug development.^209–214^

Phenotypic drug discovery (Fig. 5b) does not depend on a comprehensive understanding of disease targets but instead directly assesses the impact of drugs on biological phenotypes, including cell proliferation, apoptosis, differentiation, histological alterations, and behavioral changes in cells, tissues, or whole animal models. This approach enables the identification of drug molecules capable of producing the desired therapeutic effects.^215^

Specific screening indicators can be determined on the basis of the disease and phenotypic changes under investigation.^216–218^ These indicators can be singular, such as the cell survival rate, proliferation rate, and apoptosis rate, or they can be a combination of multiple indicators. High-content screening techniques can be employed to monitor changes in multiple phenotypic parameters simultaneously, thereby providing a more comprehensive assessment of a compound’s effects on cells or organisms.^15^

The selection of an appropriate cell model or animal model is based on the specific characteristics of the disease and the objectives of the study.^219–221^ Cell models offer the benefits of straightforward operation, low cost, and high throughput, making them ideal for large-scale compound screening. In contrast, animal models provide a more comprehensive reflection of a compound’s efficacy, pharmacokinetics, and toxicology within the entire organism.^222^

To assess the effects of compounds accurately, it is essential to establish well-designed controlled trials incorporating both positive and negative controls.^223^ A positive control typically involves a compound or treatment with a known specific phenotypic effect, which helps validate the efficacy of the screening system. Conversely, a negative control entails either the absence of compounds or the use of inert solvents to rule out the influence of nonspecific factors.^224^

During the phenotypic screening process, a substantial volume of data is generated, encompassing measurements of diverse phenotypic parameters following treatment with various compounds.^225,226^ By analyzing and statistically processing these data, it is feasible to identify compounds that exhibit significant phenotypic alterations, which are designated the screened hits.^227^

Further in-depth analysis and validation of these hits, including identification of their targets, assessment of their impact on disease-related signaling pathways, and testing for consistency and reproducibility across various model systems, are essential to determine their genuine therapeutic potential and developmental value.^228–230^

An essential subsequent step following phenotypic screening is to identify the target with which the screened compound interacts, a process known as target identification. Various techniques, including proteomics, chemical proteomics, and gene editing methods, are employed to pinpoint protein targets linked to the compound’s effects and to confirm the causal relationship between these targets and the observed phenotypic changes.^231–233^ Identifying the target will facilitate a deeper understanding of the compound’s mechanism of action, provide a foundation for subsequent drug design and optimization, and aid in the discovery of novel drug targets and biological mechanisms.

#### Drug structure optimization

By optimizing the drug’s structure, its efficacy can be enhanced, toxicity can be reduced, pharmacokinetic properties can be improved, and stability can be increased. The binding affinity with the target becomes more specific and robust, thereby enhancing the therapeutic efficacy of the drug.^234–237^

In the development of drugs, structural optimization aims to increase the drug’s cytotoxicity toward malignant cells while minimizing its toxicity to normal cells. Bay 41-4109 is classified as a first-generation heteroaryldihydropyrimidine (HAP) core protein allosteric modulator. Its further clinical development was halted because of the induction of hepatic toxicity in rats at high doses.^238^ The second generation of HAPs consists of compounds that have been structurally modified at the C2 and C6 positions on the basis of the first generation. Among these, GLS4 demonstrates superior activity, along with reduced cytotoxicity and enhanced water solubility.^239^

The chemical structure of a drug is modified to minimize its distribution and activity in nontargeted tissues or organs, thereby reducing adverse effects on normal physiological functions and enhancing the overall safety profile of the drug.^240,241^ A prodrug containing a phosphoramidate fragment can be hydrolyzed by nucleotide-binding proteins and is metabolically activated in the liver, thereby becoming a therapeutic agent that targets the liver. The prodrug design of sofosbuvir enables its accumulation in the liver, thereby enhancing its efficacy in inhibiting HCV. This innovative approach not only improves the therapeutic effectiveness of the drug but also reduces its distribution in nontarget tissues, consequently minimizing potential side effects.^242^

To optimize the pharmacokinetic properties of drugs, including their absorption, distribution, metabolism, and excretion, enhancing their bioavailability is essential to ensure that they effectively reach the site of action and maintain the appropriate concentration and duration within the body.^243^ Modifying the chemical structure of a drug to increase its water or fat solubility can significantly improve its absorption and distribution characteristics.^244^ Molnupiravir is a prodrug of *β*-*D*-*N*4-hydroxycytidine (EIDD-1931). Research has shown that while EIDD-1931 exhibits strong antiviral activity against the novel coronavirus, its structure contains multiple hydroxyl and nitrogen atoms, resulting in high hydrophilicity and rapid metabolic degradation in vivo. To address this issue, researchers esterified the hydroxyl group on the 5’-carbon of ribose with isobutyric anhydride to reduce hydrophilicity, thereby decreasing the rate of metabolism within the body and prolonging the duration of drug action. This modification led to molnupiravir, which has favorable clinical therapeutic effects.^245^

Drugs exhibit poor stability in both in vivo and in vitro environments, making them prone to degradation or failure. By undergoing structural optimization, the stability of these drugs can be significantly enhanced, thereby extending their shelf life and duration of action within the body.^246–248^ Lenacapavir, as a pioneering long-acting HIV-1 capsid inhibitor, achieves prolonged efficacy through structural optimization that incorporates electron-withdrawing groups (such as ten fluorine atoms) and enhances molecular rigidity. This strategic modification significantly improves its stability in liver microsomes. As a result of these structural enhancements, the median half-life of lenacapavir has been extended to 8--12 weeks, enabling biannual dosing and thereby realizing its long-acting potential.^249^

#### Drug synthesis

The utilization of diverse chemical reactions to modify molecular structures, thereby obtaining novel compounds with targeted biological activities, is a fundamental aspect of chemical synthesis. Common reaction types include esterification, reduction, oxidation, substitution, addition, and condensation.^250–253^ Aspirin is synthesized through the esterification of salicylic acid with acetic anhydride, a process catalyzed by sulfuric acid.^254^

Drug molecules typically incorporate specific functional groups, and through the transformation and modification of these groups, the properties and activities of drugs can be altered.^255^ Oxidizing an alcohol hydroxyl group to an aldehyde or carboxyl group or converting a carboxyl group to an ester group can modulate a drug’s water solubility, fat solubility, stability, and other characteristics.^256^

Constructing the carbon skeleton of a drug molecule is a crucial step in drug synthesis. Complex carbon skeleton structures can be assembled through carbon‒carbon bond formation reactions, including alkylation, arylation, and alkenylation reactions.^257^ These reactions facilitate the linking of various carbon fragments to progressively build the fundamental framework of the target drug molecule.^258^

The biological activity of numerous drugs is intricately linked to their stereochemical structure; therefore, controlling the stereochemistry of products during drug synthesis is essential.^259–261^ By carefully selecting reaction conditions, catalysts, or chiral reagents, the selective synthesis of the desired stereochemical configuration of drug molecules can be achieved, thereby obtaining target compounds with specific stereochemical structures. This approach not only enhances the efficacy of drugs but also minimizes side effects.^262^

The structure of drug molecules is frequently intricate, necessitating a series of chemical reactions for their synthesis. In the design of synthetic pathways, it is essential to meticulously plan the sequence, conditions, and reagents for each step to guarantee the high efficiency and selectivity of the reaction.^263,264^ Additionally, optimizing the synthetic route to increase the overall yield, minimize costs, and mitigate the environmental impact is crucial.^265,266^

Selecting the appropriate initial raw material is crucial for drug synthesis. The raw material must possess a suitable structure, functional groups, and reactivity to ensure that subsequent chemical reactions proceed efficiently.^267^ Furthermore, it is often necessary to pretreat the raw material through processes such as purification, drying, and activation to increase the effectiveness of the reaction and the quality of the final product.^268^

Reaction conditions, including temperature, pressure, solvent, and catalyst, significantly influence the rate, selectivity, and product quality in drug synthesis reactions. Optimizing these conditions on the basis of the specific reaction type and raw material properties is essential for achieving optimal reaction outcomes.^269–272^ Certain reactions require high temperatures and pressures, whereas others are highly sensitive to water or oxygen and must be conducted in anhydrous or anaerobic environments.^273^

Upon completion of the drug synthesis reaction, the product is typically subjected to separation and purification processes to achieve a high level of purity for the target compound. Commonly employed techniques for this purpose include filtration, extraction, distillation, recrystallization, and chromatography.^274,275^ These methods leverage the distinct physical and chemical properties of the substances involved to efficiently isolate the desired product from impurities, thereby increasing the quality and safety of the final pharmaceutical product.^274^

Furthermore, in the synthesis of complex structural compounds, the design of synthetic routes can be achieved through inverse synthesis analysis, a technique frequently employed in drug development, materials science, and total synthesis of natural products.^276–280^

The SYNTHIA™ platform features material inverse synthesis analysis, enabling users to access multiple synthesis pathways and the associated raw materials derived from target molecules. This is achieved by leveraging historical documents, patents, and other relevant information, ultimately leading to the identification of readily available or commercialized raw materials. Additionally, the platform supports result export and conditional screening, facilitating the design of chemical synthesis routes.^281^

#### Drug delivery

The delivery of drugs is frequently hindered by multiple biological barriers, such as physicochemical barriers (e.g., mucosal layers in the gastrointestinal tract), enzymatic degradation, and impermeability of the cellular membrane. These barriers can significantly reduce drug bioavailability and impede drug accumulation at the target site, thereby diminishing therapeutic efficacy. To address these challenges, advanced drug delivery systems, such as nanoparticle-based carriers or ligand-targeted formulations, have been developed to achieve spatiotemporally controlled drug release.^282–285^ These strategies improve drug efficacy, reduce costs, and minimize toxic side effects.^286^

The limitations imposed by biological membranes on drug delivery are among the key factors influencing the efficacy of pharmacological treatments. Biological membranes, such as cell membranes, the blood‒brain barrier, placental barriers, and intestinal mucus layers, act through physical, chemical, and biological mechanisms to restrict transmembrane transport and targeted delivery of drugs. The use of extracellular small vesicles (sEVs) to deliver antiviral siRNA combined with the modification of rabies virus glycoprotein-derived peptides (RVGs) enables targeted delivery to neural tissues. Experimental results demonstrate that sEVs-RVG can effectively penetrate both the placental barrier and the blood‒brain barrier, significantly inhibiting Zika virus infection in fetal mouse brains and alleviating symptoms associated with microcephaly.^287^

Through chemical modifications in the structure of drugs, such as altering functional groups, amino acids, or nucleic acid backbones or conjugating with known moieties or targeted ligands, the interactions between drugs and biological molecules, cells, and tissues, as well as between drugs and their target sites, are modulated to achieve controlled drug delivery within the body to fulfill the intended therapeutic functions.^288–290^ Small-molecule drug such as fentanyl can adjust their physicochemical properties by incorporating known molecular entities or directly modifying their chemical structures.^291^ Antibody‒drug conjugates, such as brentuximab vedotin, deliver highly cytotoxic drugs specifically to target antigens by binding to monoclonal antibodies.^292,293^ In nucleic acid therapy, the *N*-acetylgalactosamine (GalNAc)-siRNA conjugate givosiran increases the accumulation of siRNA in target organs and promotes cellular uptake.^294,295^ The GalNAc–siRNA conjugate can be utilized to target HBV genes, enabling liver-targeted delivery that inhibits the replication and expression of HBV. This approach offers a novel strategy for the treatment of chronic hepatitis B.^296^

Microenvironmental modulators are employed to adjust local pH levels, thereby increasing the solubility of small molecules, biologics, and nucleic acid drugs in bodily fluids, or to modify processes that hinder drug efficacy and improve therapeutic outcomes in diseased tissues.^297,298^ Osmotic enhancers, subcutaneous dispersion enhancers, and other environmental regulators are utilized to facilitate the systemic absorption of proteins and peptides. Nucleic acid drugs incorporate pH regulators along with cell-penetrating peptides and cationic lipids to improve their intracellular uptake, endosomal escape, and nuclear targeting.^299^ In cell therapy, early clinical trials of chimeric antigen receptor (CAR) T cells introduced the constitutively active IL7 cytokine receptor, which is coexpressed with a tumor-targeting CAR, to reprogram the immunosuppressive tumor microenvironment and support T-cell expansion.^300–302^

Owing to the remarkable efficacy demonstrated by CAR-T-cell therapy in numerous clinical trials involving hematologic malignancies, its safety, feasibility, effectiveness, and durability have been confirmed. On the basis of these promising outcomes, CAR-T-cell therapy is now regarded as a potential strategy for curing HIV. The initial design of CAR-T cells targeting HIV was based on CD4, which increased the likelihood of new viral particles infecting engineered cells. To address this issue, researchers have developed broadly neutralizing antibodies (bNAbs) to replace CD4 as the antigen recognition domain in the CAR structure. Preventing CAR-T-cell infection is crucial for maintaining CAR-T-cell persistence and anti-HIV activity. By employing gene editing techniques to knock out the CCR5 gene, it is possible to prevent CAR-T-cell infection and confer permanent resistance to HIV infection. The feasibility of using bNAb-based CAR-T cells for treating individuals with *CCR5∆32* deletion was tested, and the results indicated that CCR5-modified CAR-T cells exhibited superior control over viral replication.^303^

With the assistance of various pharmaceutical formulations, including tablets, capsules, ointments, solutions, hydrogels, and polymer implants, drug delivery systems integrate controlled release mechanisms such as dissolution, diffusion, penetration, and ion exchange. These mechanisms, combined with drug modification and environmental adaptation, physically protect drugs from adverse environmental effects and achieve controlled release for enhanced therapeutic outcomes.^304,305^ Common drug delivery carriers include both artificial and natural carriers. Artificial carriers, such as liposomes, polymer carriers, micelles, and other nanoparticles, can be further optimized through surface modifications and the incorporation of stimulus-responsive functionalities.^306^ In 2021, two novel mRNA vaccines for the coronavirus, BNT162b2 and mRNA-1273, received regulatory approval for market release. These vaccines utilize lipid nanoparticles to encapsulate mRNA molecules, thereby protecting them from degradation by nucleases and facilitating their entry into cells. This innovative delivery system not only addresses the challenges associated with the difficulty of mRNA crossing cell membranes and its susceptibility to nuclease degradation in vivo but also plays a crucial role in ensuring the stability and efficacy of mRNA vaccines.^307^

In the field of drug delivery, numerous challenges persist, including nanoparticle-induced toxicity, off-target immune responses, instability during storage or systemic circulation, and manufacturing scalability limitations. Addressing these issues requires a deep interdisciplinary approach involving materials science, bioengineering, and clinical medicine. The core challenge lies in how to deliver drugs precisely and efficiently to the target while ensuring safety.

The size of nanoparticles (such as liposomes and polymeric nanoparticles), typically ranging from 10 to 200 nm, can interact with biological barriers in ways that may induce toxicity. Additionally, issues such as material toxicity and abnormal biodistribution further complicate the situation. To address this issue, nontoxic carriers such as human serum albumin can be utilized to reduce toxicity. In light of the rapid clearance of nanoparticles by macrophages, lipid nanoparticle (LNP) systems utilizing ionizable lipids offer precise regulation of endosomal escape.^308^ Furthermore, stability can be enhanced through techniques such as dual encapsulation and dynamic covalent bonding.^309,310^ Continuous flow production presents a promising solution for alleviating large-scale manufacturing challenges in this domain.^311^ The rapid degradation of mRNA can be addressed through chemical modification techniques and carrier encapsulation methods for protection.^312,313^ The off-target effects associated with targeted delivery can be regulated by corresponding technologies, such as pH modulation and near-infrared light.^314,315^

The METiS pharmaceutical AI-driven drug delivery platforms include the AI-driven nucleic acid delivery system design platform (AILNP), the AI-driven mRNA sequence design platform, and the AI-driven small molecule preparation design platform. Notably, AILNP represents the world’s first high-throughput automated LNP design platform. It integrates large-scale models and generative AI technologies to develop lipid language models and lipid generation models, construct a lipid library of millions of compounds, and facilitate the development of multiorgan targeted nanomaterials via molecular simulation techniques.^316^

## Structure-guided antiviral drugs

Target discovery plays a pivotal role in drug development, serving as the initial step for TBDD. If we envision the disease as a lock, then the target represents its core mechanism. By identifying and scrutinizing the three-dimensional (3D) structure of this lock core, we can subsequently develop a tailored key that aligns with its unique structural characteristics–herein lies the essence of drug development.

Once the antiviral target is identified, structure-based drug design can be conducted. This involves the use of molecular docking and virtual screening techniques to rapidly identify candidate molecules that may bind to the target protein from a large pool of compounds. Through analysis of the interaction between the drug and the target protein, the structure of the lead compound can be optimized to enhance its affinity, selectivity, and pharmacokinetic properties. Designing drugs that can bind to multiple sites on the target protein helps reduce the development of viral resistance. Additionally, by targeting conserved or cross-species targets in viruses, structure-based drug design facilitates the development of broad-spectrum antiviral drugs that are effective against multiple viruses.

The selection of antiviral drug targets typically focuses on conserved sites to facilitate the development of therapeutic vaccines or drugs with broad-spectrum efficacy and safety. Virus-targeted inhibitors aim to impede distinct stages of the virus life cycle, such as viral entry (S inhibitors), proteolytic processing (M^pro^ inhibitors and PL^pro^ inhibitors), RNA synthesis (blockers of NSP12 to 16), and virion assembly (N inhibitors), in the case of SARS-CoV-2.^25^

In addition to the conventional protein targets mentioned above, certain genetic regions can be utilized for therapeutic intervention. The structural and functional regulation of HIV-1 depends on the number of 5′-guanosines, which are located within an untranslated genomic region characterized by a low mutation rate. Consequently, controlling the 5′-guanosine site represents a promising approach for addressing the challenge posed by HIV drug resistance.^317^

The advantages of TBDD include rapid throughput, increased efficiency, and an edge in the approval of follower-tracking drugs. However, challenges exist, such as the limited number of available targets. The human genome comprises approximately 20,000 genes. While approximately 4500 of these genes are considered part of the druggable genome, currently, fewer than 700 of them have been targeted by drugs approved by the FDA.^318^ This scarcity means that pharmaceutical companies must compete for these few viable options. In addition, the success rate is relatively low, as most of the screened compounds fail to reach their intended proteins after entering the body. In light of the shortage of drug targets, alternative mechanisms such as targeting protein degradation (TPD) and modulating biomolecular condensates offer promising opportunities for the development of first-in-class antiviral drugs.

The viral proteins are often in a state of dynamic conformational changes (such as the dimerization of the HIV protease and the conformational transitions of the influenza virus hemagglutinin).^319^ Static crystal structures may not comprehensively reflect their functional states, leading to deviations in predicting drug binding sites. Rapid mutations in viruses (such as HIV, HCV, and SARS-CoV-2) can result in alterations to target amino acid sequences, potentially rendering drug binding pockets ineffective due to these mutations. These factors significantly limit the development of structure-based antiviral drug discovery. The HIV fusion inhibitor enfuvirtide works by binding to HIV gp41 to inhibit membrane fusion; however, it requires injection for administration, is costly, and is prone to resistance due to viral mutations, which restricts its clinical application.^320^ Similarly, monoclonal antibodies targeting the SARS-CoV-2 S protein (e.g., bamlanivimab) rapidly lose neutralizing activity due to mutations in the S protein associated with variants such as Omicron (including N501Y and E484K).^321^

In the face of these challenges, structure-based antiviral drug discovery must integrate techniques such as cryo-electron microscopy and molecular dynamics simulations to elucidate the structures of viral proteins in various functional states, thereby facilitating the development of conformation-selective drugs. Targeting multiple stages of the viral life cycle (such as entry, replication, and assembly) through multitarget drug design can help mitigate the risk of resistance. The application of AI algorithms (e.g., AlphaFold for protein structure prediction) can accelerate drug discovery and overcome the limitations associated with traditional virtual screening methods.

### Chemical libraries

The quality of chemical libraries significantly impacts the reliability, reproducibility, and success of high-throughput screening. Therefore, it is crucial to invest in high-quality libraries that cover diverse chemical spaces, validated biological activities, and rigorous quality control measures to maximize the likelihood of identifying novel drug candidates.

A wide array of compound libraries, such as diverse commercial compound libraries, FDA-approved drug libraries, known activity libraries, target compound libraries, natural product libraries, and fragment libraries, exist. Each of these distinct types of compound libraries possesses unique characteristics and applications, thereby offering researchers an extensive selection.

Commercial compound libraries from ZINC, PubChem, DrugBank, ChEMBL, ChemDB, HMDB, BindingDB, and SMPDB offer a diverse array of compounds with distinct structural features that facilitate the exploration of novel and biologically active molecules (Table 2). The FDA-approved drug library comprises marketed drug entities, which enables researchers to investigate their pharmacological effects and clinical applications. The known activity library contains compounds with well-defined biological activities that can significantly contribute to the advancement of new drugs or therapies. The target compound library is specifically screened against biological targets to increase the efficiency and success rate of drug development endeavors. The natural product database primarily encompasses naturally occurring compounds with potential biological activity and medicinal value. A fragment library consists of smaller molecular fragments that can be utilized for constructing and optimizing novel drug molecules.

**Table 2:** An introduction to compound libraries

Name	Scale	Properties	Access methods
ZINC	750 million compounds	Provides the structure of the compound, important properties (e.g., xlogP, solubility, etc.) as well as 2D and 3D structure, supplier information.	https://zinc15.docking.org/
PubChem	119 million compounds	Standardized chemical structure data and biological activity data of compounds, such as half inhibitory concentration (IC_50_), are provided.	https://pubchem.ncbi.nlm.nih.gov/
DrugBank	500,000 drugs	It provides information on the chemical structure, pharmacological characteristics, transporter-related information, drug side effects, and so on.	https://go.drugbank.com/
ChEMBL	2.49 million compounds	It collects pharmacochemical data and knowledge in the drug research and development process.	https://www.ebi.ac.uk/chembl/
ChemDB	5 million compounds	Includes predictions, or experimentally determined physico-chemical properties of chemical substances, such as 3D structure, melting temperature, and solubility.	https://cdb.ics.uci.edu/
HMDB	220,945 metabolites	The database contains detailed information on small molecule metabolites found in the human body.	https://hmdb.ca/
BindingDB	1.3 million compounds	Focuses on the interaction of proteins of drug targets with small drug-like molecules. Contains binding data, protein targets, and small molecules.	https://www.bindingdb.org/rwd/bind/index.jsp
SMPDB	30,000 molecule pathways	Contains multiple small molecule pathways discovered by humans, specifically designed to support pathway elucidating and pathway discovery in metabolomics, transcriptomics, proteomics, and systems biology.	https://smpdb.ca/

Compound libraries sourced from various brands and suppliers, such as Chemdiv, Enamine, Lifechemicals, Specs, Chembridge, Maybridge, Microsource, Vitas-M, and Interbioscreen, may offer distinct advantages and characteristics. Therefore, a comprehensive evaluation should be conducted when selecting a compound library to align with the research needs and objectives.

In addition to commercially available compound libraries, researchers have the option to construct their own chemical libraries suited to particular areas of interest.

#### Readily accessible (REAL) screening compounds

**Fig. 6:**
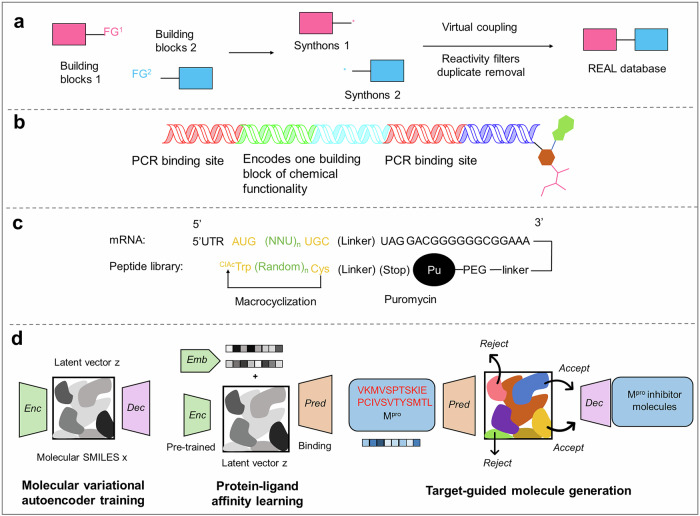
Several approaches to the generation of chemical libraries. **a** The general principle of REAL database generation via one-step, two-component reactions. **b** Structural composition of a DEL molecule. **c** An mRNA-displayed library created via genetic code reprogramming and spontaneous posttranslational cyclization. **d** Molecular variational autoencoder (VAE) training on large-scale chemical SMILES (x) data and mapping of existing protein–ligand affinity relationships on the VAE latent space (z) by training a binding predictor. For the latter, a pretrained neural network utilizing a large number of protein sequences is embedded. Samples are generated from the model of VAE latent vectors via guidance from a set of molecular property predictors (e.g., protein binding). For a given target protein sequence, z sampled vectors corresponding to strong target binding affinity are accepted, whereas vectors corresponding to weak target binding affinity are rejected. The accepted z vectors are then decoded into molecular SMILES

Yurii S. Moroz et al. utilized REAL starting materials and employed two- or three-step three-component reaction sequences with previously verified chemical reactivity, making it feasible to generate a vast virtual library of approximately 29 billion compounds (Fig. 6a). By applying predicted physicochemical descriptor values, this generated chemical space encompasses a majority of similar drugs and “beyond rule-of-five” members; however, there still exists a subset exceeding 22 million compounds that adhere strictly to Churcher’s rules for lead similarity.^322^

#### DNA-encoded chemical library (DEL) technology

DEL tags chemical molecular primitives with predesigned oligonucleotide sequences, constructing a DNA-encoded compound library through combinational chemistry principles (Fig. 6b). The screening strategy involves incubating the DEL with a target protein, removing unbound, nonspecific molecules on the basis of their binding affinity to the protein, and achieving significant enrichment of specific binders with strong affinity through different elution methods. The compound identity can be decoded according to the DNA tag chemically conjugated to the small molecule by polymerase chain reaction (PCR) amplification and next-generation sequencing analysis. Damian W. Young et al. utilized this method for the rapid screening of high-affinity binders and the discovery of SARS-CoV-2 M^pro^ inhibitors, identifying compounds with low nanomolar *K*_i_ values.^323,324^

Additionally, this technique was utilized by Matthew D. Disney and colleagues for the screening of monomeric ribonuclease (RNase) L binders, ultimately providing a compound that induces dimerization of RNase L.^325^ The resulting compound was incorporated into the design of next-generation ribonuclease-targeting chimeras. Accordingly, DEL can be used to identify compounds that bind and recruit effector proteins to heterobifunctional compounds.

#### Macrocyclic peptide library

The random nonstandard peptide integrated discovery (RaPID) platform enables efficient screening of macrocyclic peptides with preferential binding affinity toward a target.^326^ The RaPID was prepared via a flexible in vitro translation system facilitated by flexible tRNA-aminoacylating ribozymes (named flexizymes), along with mRNA display technology. To construct mRNA libraries for rapid screening, the UAG stop codon is incorporated to bind the 3’ end of each mRNA to the puromycin linker. This results in the puromycin portion attaching to the C-terminus of the translated peptide, creating a covalently linked peptide “phenotype” corresponding to the mRNA “genotype” (Fig. 6c). Affinity-driven screening of peptide‒mRNA fusion libraries is conducted for specific target proteins immobilized on magnetic beads. The mRNA bound to the isolated peptide is subsequently amplified through reverse transcription and PCR, followed by transcription into the next round of the mRNA library. By iteratively performing multiple rounds of these procedures, mRNAs encoding peptides with strong binding affinity are enriched, and their sequences can be determined via next-generation sequencing of the corresponding cDNA libraries. Hiroaki Suga et al. also utilized this approach in the screening of SARS-CoV-2 M^pro^ inhibitors.^327^ One resulting potent M^pro^ inhibitor (IC_50_ = 50 nM), GM4, exhibited a 5.2 nM dissociation constant.

#### Generative model utilizing deep learning

De novo molecular design, which involves the proposal of previously unidentified compounds with desired properties, poses a formidable challenge in drug discovery and materials engineering. One of the primary reasons for the low success rate is the vast search space, estimated to encompass 10^33^–10^80^ viable molecules, among which only a small fraction typically possesses the sought-after characteristics. Consequently, it is impractical to select compounds individually through experimentation. Deep learning generative models hold promise in discovering novel molecules with desired functions in an exploratory manner by first acquiring dense and continuous representations of known chemicals and subsequently manipulating latent vectors to decode unseen molecules. Such models offer an opportunity to venture into uncharted chemical spaces without being constrained by human bias.

The CogMol framework (Fig. 6d), a deep generative framework, was developed by Payel Das et al. utilizing extensive datasets of chemical molecules, protein sequences, and protein‒ligand binding interactions. It serves as a foundational model for the molecular design of target-sensing inhibitors without requiring detailed fine-tuning with target-specific data. Moreover, it demonstrates the ability to extrapolate to target sequences not included in the original training data. Consequently, the broad versatility of the CogMol framework positions it within the emerging category of foundation models that are pretrained on large amounts of unlabeled data and can be readily adapted for various downstream tasks with minimal fine-tuning.^328^

The researchers conducted the first experimental validation of the broad utility and readiness of the CogMol deep generation framework by synthesizing and testing a series of compounds designed to inhibit the SARS-CoV-2 S protein and M^pro^. The designed inhibitors targeting the S protein demonstrated potent antiviral activity against the variant at micromolar levels, thereby further establishing the potential of the deep generative framework to expedite and automate hit discovery cycles.^328^ This process, known for its low yields and high attrition rates, also contributes to advancing the scientific understanding of less explored drug targets.

### Compound screening

Hit identification is the first step in a drug discovery project. For several decades, high-throughput virtual screening and fragment-based screening have facilitated the discovery of antiviral hit compounds. In addition, to advance hit compounds into more drug-like lead compounds, a preliminary evaluation of drug similarity is conducted during the compound screening process by establishing a discriminant model or a multiparameter scoring platform.

#### Virtual screening

The commercial compound library is experiencing rapid growth, with over 10 billion REAL molecules currently offered by chemical suppliers.^322^ This presents an opportunity for the discovery of novel therapeutic drugs, as virtual screening can play a pivotal role in identifying potential molecules when combined with high-resolution target crystal structures and extensive compound libraries. By employing a structure docking function to evaluate large compound libraries, it is possible to rapidly screen top-ranked compounds with potential binding capabilities. These compounds can subsequently be tested and optimized, significantly enhancing the efficiency of lead compound discovery.

**Fig. 7:**
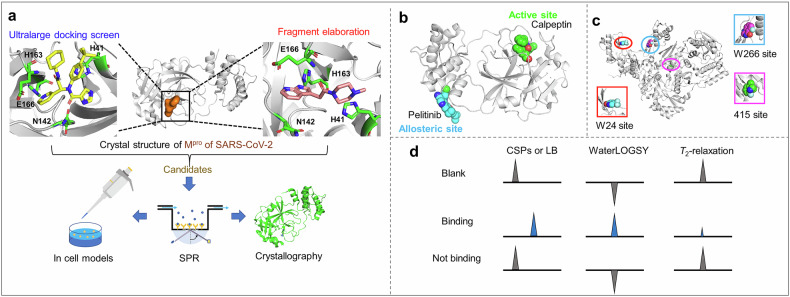
Several approaches to compound screening. **a** Overview of virtual screening approaches. Two virtual screening strategies were employed to identify inhibitors of the main protease of SARS-CoV-2 from ultralarge chemical libraries. The predicted inhibitors were subsequently evaluated through biophysical and biochemical assays, crystallography, and models of virus-infected cells. **b** X-ray crystallographic screening identifying compounds that target active and allosteric sites. **c** HIV-1 RT-rilpivirine with bound Halo library fragments. **d** Schematic representation of NMR-assisted fragment screening experiments and criteria used to identify binders and nonbinders

In the early stages of a viral outbreak, there is a dearth of potent treatment options. Virtual screening has emerged as a potent strategy for identifying potential antiviral drugs against this pathogen. During research, multiple rounds of screening are often imperative to discover efficacious small-molecule compounds. Typically, in the initial screen, extensive databases are scrutinized, and compounds exhibiting promising scores are selected for subsequent testing. An additional round may be conducted with a focus on optimizing a fragment identified through crystallographic screening.^329,330^ The screened compounds were then validated via cell models to identify potential inhibitors (Fig. 7a). Through the application of screening and compound optimization techniques, Jens Carlsson et al. identified a potent broad-spectrum inhibitor that has significant activity (EC_50_ = 77 nM) against M^pro^.^331^ Similarly, in the exploration of an inhibitor of NSP14 *N*7-methyltransferase (*N*7-MTase), Brian K. Shoichet et al. identified five compounds with IC_50_ values below 10 μM through virtual screening.^332^

After virtual screening, the most promising compounds undergo direct testing through a high-throughput X-ray crystallographic screen. This approach not only enables the identification of compounds that target the active site but also facilitates the discovery of compounds that bind to allosteric sites (Fig. 7b).^333^

In addition to screening extensive compound libraries, the exploration of previously approved drugs on the market has emerged as a strategic approach in antiviral research.^333^ The incorporation of approved drugs or those undergoing clinical trials into the screening process of drug-like compound libraries enhances the efficiency of hit compound discovery. Revisiting established drugs through novel investigations can expedite research and development while simultaneously benefiting a larger patient population. William L. Jorgensen et al. utilized this strategy, which resulted in the identification of five drugs with IC_50_ values below 40 μM.^334^

In addition, remdesivir,^335,336^ which has been utilized in the study of treatments for Ebola virus and various RNA virus infections, has received approval from the FDA as a broad-spectrum antiviral agent. This marked its distinction as the first officially sanctioned treatment for novel coronavirus infections. This approach, known as drug repositioning, involves repurposing drugs that are already available on the market or are in development for new medical uses. By leveraging safety and efficacy data from existing drugs, this strategy can significantly reduce the time, cost, and risk associated with new drug development. Notable examples of successful retargeting include sildenafil (initially developed to treat angina pectoris before being repurposed as an erectile dysfunction drug) and zidovudine (originally a chemical drug before becoming the first FDA-approved anti-HIV medication).^337^

#### Fragment-based screening

Compared with screening commercial drug-like compound libraries (>300 Da), screening fragment molecules (∼60–300 Da) allows the exploration of novel binding spaces within binding sites. By combining fragments from different locations, new small-molecule compounds with unique structures and enhanced activity can be discovered, offering potential for drug development.^338^ By utilizing a relatively small set of compounds, Fandi Sutanto et al. explored a wide range of chemical spaces, thereby facilitating the identification of novel drug-like compounds and potential drug candidates. Moreover, high-throughput synthesis permits the rapid acquisition of target compounds in substantial quantities for experimental evaluation, thereby accelerating the drug development process.^339^

Certainly, when multiple sites with “universal fragments” (such as the Halo library) are targeted,^340^ Dávid Bajusz et al. incorporated halogenated fragments into screening procedures, which facilitated the identification of hot spots and cryptic sites.^341^ Key interactions between fragments and proteins can be elucidated through nuclear magnetic resonance (NMR), surface plasmon resonance (SPR), thermal shift assays and X-ray crystallography, enabling the identification of fragments that serve as valuable starting points for structure-guided drug development.^342^ This approach holds great promise for combating future viral threats.^343^ Joseph A. Newman et al. discovered two novel binding sites for HIV-1 RT and identified several key hotspots (Fig. 7c). The subsequent design of compounds aimed at targeting these newly identified sites will facilitate the discovery of novel antiviral drugs.

In addition to SPR and X-ray crystallography, NMR-assisted fragment screening is one of the most widely employed techniques for fragment-based drug discovery. It encompasses the following experimental procedures: chemical shift perturbation (CSP) or line broadening (LB), waterLOGSY and *T*_2_-relaxation experiments (Fig. 7d). Harald Schwalbe et al. successfully utilized a variety of 1D ^1^H-NMR binding assays to identify hit molecules that bind to RNA.^344^ On the basis of the identified hits, the key functional groups that effectively target RNA were obtained. This laid the groundwork for optimizing the compound’s structure.

### Structure-guided compound optimization

#### Structural biology-oriented chemical structure optimization

After conducting a preliminary screening of a potential hit compound, it becomes imperative to optimize its structure via various synthetic strategies (such as bioisosterism, molecular hybridization, and scaffold hopping) to effectively increase its activity and improve its drug-like properties. As previously described, employing an established drug approach led to the identification of 14 inhibitors against M^pro^ from a virtual screen encompassing approximately 2000 known and approved drugs. The IC_50_ range of perampanel is 100–250 μM because of interference between the fluorescence of the compound and the assay products. However, its relatively simple structure allows for facile synthesis of analogs and shows remarkable docking results. Therefore, perampanel was chosen for structural optimization.^334^

**Fig. 8:**
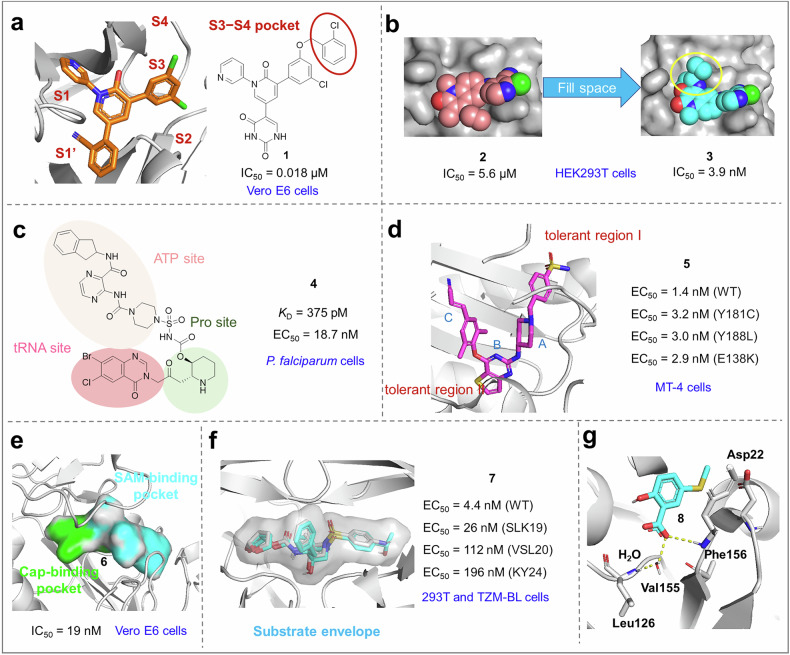
Various structure-guided compound optimization strategies. **a** Structural biology-oriented structure optimization of perampanel.^345^ **b** Application of the shape complementation strategy in the optimization of compound **3**.^346^ **c** Potent activity of compound **4** achieved by occupying multiple sites.^348^ **d** Molecule inhibitors of resistant HIV-1 strains developed by interactions with solvent-exposed regions.^351^ **e** Bisubstrate inhibitor consisting of two covalently linked fragments.^355^ **f** Optimization of the structure of darunavir according to the substrate envelope hypothesis.^357^ **g** Utilization of solvent networks in ligand design

William L. Jorgensen et al. used docking studies to reveal that perampanel predominantly occupies the S1, S1’, and S2 pockets, thereby facilitating an analysis of its binding mode (Fig. 8a). With the assistance of free-energy perturbation (FEP) calculations, substituents were introduced into the S3 − S4 pocket to generate a series of derivatives. Among them, compound **1** significantly inhibited M^pro^, with an IC_50_ of 18 nM.^345^

In the context of structure-guided design, shape complementarity emerges as a highly effective strategy. By specifically targeting a subpocket adjacent to Val18 (Fig. 8b), B-cell lymphoma 6 protein inhibitors were meticulously engineered by Swen Hoelder et al. to achieve a more than 300-fold increase in activity (compound **3**) compared with lead compound **2**.^346^ The inability to form stronger polar interactions prompted the adoption of a ring closure strategy, resulting in more compact conformations. By exploring rings of varying sizes, a conformationally constrained core was identified that maximized binding space utilization, yielding a highly efficient inhibitor.

#### Targeting novel or multiple sites

Targeting novel sites or multiple sites for occupancy of additional binding pockets represents an effective approach to augment the binding affinity of compounds. By specifically targeting novel sites or multiple sites, drugs can act more precisely on the intended target, thereby reducing potential side effects and enhancing the effectiveness of treatment. Moreover, drugs targeting novel sites or multiple sites may circumvent existing resistance mechanisms and offer novel strategies for treating drug-resistant viruses.

This tactic was employed during the optimization process from perampanel to compound **1**, as mentioned above.^345^ The introduction of a benzophenol substituent led to a significant increase in activity, with an improvement of over 300-fold (Fig. 8a). In addition, our group has made significant progress in the design of compounds targeting the 150 cavities of influenza virus NAs, resulting in improved compound resistance.^347^

In recent years, aminoacyl-tRNA synthetases, including prolyl-tRNA synthetase (ProRS), have emerged as promising targets for malaria chemotherapy. The active site of ProRS comprises three distinct pockets that specifically bind ATP, proline, and tRNA (Fig. 8c). Halofuginone, one of the most potent known antimalarial drugs, is a synthetic derivative of the natural product febrifugine. However, its poor tolerance and unknown mechanisms of action in both hosts and parasites have impeded drug development efforts. Unlike halofuginone and its analogs, a novel class of ProRS inhibitors exemplified by T-3767758 interact with the ATP-binding pockets adjacent to the active site. Ralph Mazitschek et al. successfully identified various linkers and substituents, leading to the development of triple-site inhibitors (**4**). These multisite inhibitors exhibited significantly improved affinity over single-site binders by orders of magnitude, thereby validating the feasibility of this strategy.^348^

In addition to their active sites, certain specialized structures also possess potential as drug targets. HIV-1 NCp7 is a two Cys_2_HisCys zinc knuckle protein (N-Zn and C-Zn) that plays a pivotal role in viral replication. The C-Zn knuckle undergoes transitions between its primary state (zinc coordinated by three cysteines and one histidine) and two folded morphisms, wherein one of the coordination bonds (Cys413-S*γ*-Zn or His421-N*ε*2-Zn) is hydrolyzed. Consequently, G. Marius Clore et al. designed thioester compounds (*S*-(2-((3-amino-3-oxopropyl)carbamoyl)phenyl) pent-4-ynethioate) that can selectively disrupt the C-Zn knuckle by acylating Cys413, providing a solid foundation for rational drug design.^349^

#### Exploiting solvent-exposed regions

Solvent-exposed regions or solvent-filled pockets within or near the ligand-binding sites of drug target proteins present promising opportunities for substantial modification of existing small drug molecules with minimal loss of activity. By focusing on solvent exposure regions, structural optimization of compound molecules can enhance drug affinity and selectivity, improve drug solubility and bioavailability, reduce drug side effects, and overcome drug resistance.^350^

The introduction of highly active antiretroviral therapy in the mid-1990s, comprising drug cocktails containing nucleoside RT inhibitors, nonnucleoside RT inhibitors, and/or protease inhibitors, led to a significant reduction in HIV/AIDS morbidity and mortality by effectively suppressing viral replication and disease progression. Crystallographic studies have revealed that the nonnucleoside RT inhibitor binding pocket (NNIBP) includes solvent-exposed regions, such as tolerant regions I represented by the Pro236 hairpin loop, as well as tolerant regions II encompassing the entrance channel characterized by a large open region preceding Glu138 (Fig. 8d).^351^

Scaffold decoration focusing on specific nonpolar interactions has gained increasing recognition as a valuable approach for the discovery of NNRTIs. Our research team introduced a variety of substituted benzyl-linked piperidin-4-yl-amino moieties to replace the aromatic *p*-cyanoaniline moiety (A-ring) in etravirine. The nitrogen atom of the piperidine ring (**5**) could interact with Lys103, thereby enhancing the resistance distribution. The substituted benzyl group was oriented toward tolerant region I to facilitate interaction with NNIBP. Furthermore, piperidine-substituted thiophene[3,2-*d*]pyrimidine derivatives were employed to modify the pyrimidine (B-ring) of the compound etravirine. Compound **5** exhibited superior potency after multiple optimizations. Compared with etravirine, it demonstrated a threefold increase in antiviral efficacy against wild-type (WT) HIV strains and five- to sevenfold greater activity against Y181C, Y188L, E138K, and F227L/V106A mutants.^351^

#### Substrate structure

In the field of drug design, competitive inhibitors have been designed through the modification and optimization of the structure of natural substrates. In addition to direct optimization, strategies involving double substrate binding and substrate envelope hypotheses have also been developed. The residues that come into contact with inhibitors outside the substrate envelope can mutate without affecting substrate recognition, resulting in resistance. In contrast, inhibitors that are well adapted to the substrate envelope take advantage of the evolutionary constraints of substrate recognition. Therefore, designing powerful protease inhibitors that adapt to the substrate envelope framework will be less likely to lead to drug resistance.^352^

A bisubstrate inhibitor comprises two covalently linked fragments, each binding to a substrate or cofactor element, potentially mimicking the ternary transition state of a bireactant-catalyzed reaction.^353^ The SARS-CoV-2 NSP14 *N*7-MTase, which is responsible for transferring the methyl group from the *S*-adenosyl-_L_-methionine (SAM) cofactor to the *N*7-guanosine cap, is a prime candidate for this bisubstrate binding strategy (Fig. 8e). Françoise Debart et al. designed a series of nucleoside-derived inhibitors, among which compound **6** demonstrated exceptional inhibitory activity in the double-digit nanomolar range against the *N*7-MTase NSP14.^354,355^

It has been postulated that inhibitors conforming to the substrate envelope exhibit a superior resistance profile by safeguarding against binding site mutations, which would otherwise impede access to the substrate envelope and significantly compromise natural substrate binding.^356^ Currently, this hypothesis is extensively employed in the design of HIV protease inhibitors. Using this approach, Akbar Ali et al. optimized the structure of darunavir by introducing a 4-(1-hydroxyethyl)phenyl moiety, resulting in the formation of compound **7**. Notably, compound **7** exhibited favorable activity against both WT and multidrug-resistant HIV-1 variants (Fig. 8f) while maintaining its potency.^357^

#### Electrostatic interactions

Electrostatic interactions between molecules and receptors are essential for molecular recognition and influence binding free energy. Therefore, assessing the electrostatic complementarity of protein‒ligand complexes offers valuable insights into ligand binding mechanisms and strategies to increase binding affinity. Ideally, optimizing their electrostatic potentials at the protein‒ligand interface should maximize mutual complementarity while minimizing any unfavorable dissolution effects.^358^

The receptor-binding domain (RBD) of SARS-CoV-2 has a significantly larger positively charged region on its surface.^359^ Electrostatic interactions are crucial in the process of viral invasion, serving as the primary mechanism for high-charge anionic inhibitors. Consequently, Rainer Haag et al. designed negatively charged polysulfate that can effectively bind to the S protein through electrostatic interactions. Among these inhibitors, linear polyglycerol sulfate (LPG_20_S_0.94_, degree of sulfation determined via elemental analysis) exhibits optimal inhibitory activity, with an IC_50_ value of 67 mg mL^-1^.^360^

#### Investigate water binding sites within ligand binding pockets

Water molecules are found within protein channels and are frequently associated with ligands in protein crystal structures, underscoring their relevance. Water-mediated interactions, particularly hydrogen bonding, are sometimes a prerequisite in the process of drug binding.^353^

The macrodomain of NSP3 from SARS-CoV-2 is responsible for the removal of adenosine diphosphate ribosylation posttranslational modification and is vital for the ability of the virus to evade the immune system. James S. Fraser’s group analyzed the binding structure of the fragments (**8**, Fig. 8g) and reported that most water molecules within the adenosine site network can either participate in bridging interactions or be displaced. This analysis highlights the potential utilization of solvent networks in ligand design, either by introducing extended groups that bridge hydrogen bond networks or through targeted displacement of water molecules.^361^

### Covalent binding strategy

Covalent inhibitors are a class of small-molecule compounds that form stronger bonds with the amino acid residues of specific proteins through covalent interactions, thereby increasing their inhibitory activity. However, researchers still have concerns regarding the potential off-target effects and nonspecific protein modifications associated with covalent inhibitors. To address these challenges, Nir London et al. developed the computational pipeline “Covalentizer” to identify irreversible inhibitors on the basis of the structure of a noncovalent conjugate.^362^ The pipeline consists of four consecutive steps: fragmentation, electrophile diversification, covalent docking, and root-mean-square deviation (RMSD) filtering. Molecules are broken down through the synthesis of accessible bonds. A potential library of electrophilic analogs, consisting of several hundred compounds, is generated for each substructure. The protein structure is then docked with the appropriate analog library via all available cysteine rotors. For each docking compound, the RMSD between the maximum common substructure of the reversible compound and the covalent analog was calculated.

**Fig. 9:**
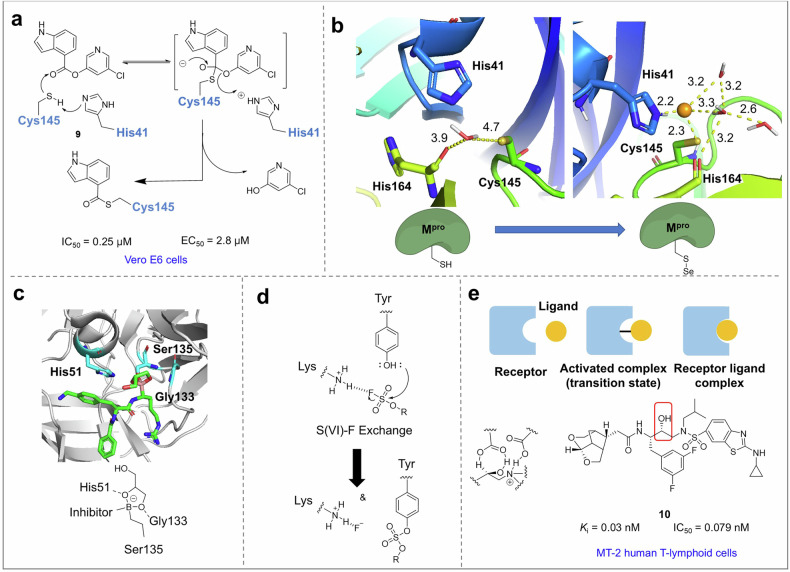
Representative examples of covalent inhibitors and transition state analogs. **a** Mechanism of the inhibition of SARS-CoV-2 M^pro^ by compound **9**.^363^ **b** Hydrolysis of the ebselen derivative by M^pro^, leaving selenium (Se) bound to Cys145. **c** Boric acid compound located in the substrate-binding site of the West Nile virus NS2B-NS3 protease. **d** Schematic representation of the SuFEx reaction and its requisites. **e** Enzymes with significantly greater affinities for transition state structures (red frame).^375^

In addition to conventional covalent warheads that directly form covalent bonds with amino acids, small molecules may also break bonds while forming new bonds. Arun K. Ghosh et al. designed compound **9**, which exhibited an inhibitory IC_50_ value of 250 nM against SARS-CoV-2 M^pro^ in Vero E6 cells and an antiviral EC_50_ value (antiviral activities) of 2.8 μM. The X-ray crystal structure revealed that after forming a covalent bond with M^pro^, the compound subsequently broke the intramolecular bond (Fig. 9a).^363^ This finding presents a novel concept for designing covalent inhibitors, thereby offering valuable insights for future drug development. Similarly, Atul Kumar et al. designed an ebselen analog that exhibits a comparable mechanism. (Fig. 9b)^364^.

Covalent inhibitors possess distinct advantages in enhancing compound binding affinity. Christian D. Klein et al. designed a peptide inhibitor that incorporates boric acid fragments to interact with a flavivirus protease, leading to a significant thousand-fold increase in affinity (Fig. 9c).^365^ This study contributes to the elucidation of the underlying mechanism governing compound binding, facilitating research in the development of novel therapeutic interventions.

Click chemistry serves as a potent tool in proteomic applications and drug discovery. Covalent compounds can be designed by employing click reactions to form covalent bonds. One category of clickable molecules includes those capable of undergoing sulfur(VI) fluoride exchange (SuFEx). Through screening a library of fluorosulfate compounds amenable to SuFEx reactions, Stefan G. Sarafianos’s group identified the fluorosulfate compound (Fig. 9d) as a potent inhibitor of the HIV-1 CA protein. This compound forms a covalent bond with CA via a SuFEx reaction at Tyr145 and exhibits antiviral activity in a cell culture system by perturbing virus production.^366^

Allosteric sites, along with orthosteric sites, can be strategically designed for covalent inhibitors. Tarun M. Kapoor et al. employed “scout fragments” and a function-first approach combined with enzyme analysis via conformation probe pairs and mass spectrometry (MS). This led to the development of a selective covalent inhibitor that specifically interacts with the allosteric site of the SARS-CoV-2 NSP13.^367^ These findings suggest a method for identifying potential covalent inhibitors and druggable allosteric sites in conformationally dynamic mechanoenzymes.

Furthermore, the design of heterobifunctional molecules, including covalent inhibitors and PROTACs, can promote interactions between proteins of interest and ubiquitin ligases, leading to selective protein degradation.^368^

Irreversible covalent inhibitors, which include electrophiles such as acrylamide, *α*-haloketones, epoxides, azopropyl rings, vinyl sulfones, and activated acetylene, possess favorable pharmacokinetic/pharmacodynamic characteristics; however, their use is often limited due to their nonselective covalent reactivity and the associated risk of adverse reactions. To increase the selectivity of covalent inhibitors, the alkyne moiety is incorporated as an electrophilic reagent. Alkynyl inhibitors exhibit specific thiol reactivity and effectively inhibit target protein activity by forming irreversible covalent bonds with catalytic cysteine residues.^369^ Moreover, the attachment of electrophilic reagents directly to the reversible inhibitor scaffold enables the targeting of solvent-exposed cysteines located more than 10 Å from the binding site.^370^

The use of irreversible covalent inhibitors may restrict their application in scenarios where the ability to revert to prototypes is essential. In other words, their nonreversible nature can restrict their application in scenarios requiring temporary inhibition. On the other hand, reversible covalent inhibitors, which typically include electrophiles such as aldehydes, activated ketones, *α*-ketoamides, nitriles, and boric acid derivatives, form reversible covalent bonds with their target. As a result, they offer pharmacokinetic properties that position them between irreversible inhibitors and noncovalent inhibitors while mitigating the risks associated with off-target effects.^371^

### Antiviral drug design via transition state analogs

In the process of enzyme catalysis, one or more transition state structures are encountered, wherein the energy barrier forming a high-energy transition state governs the reaction rate. Compared with stable states such as substrates, intermediates, or products, the enzyme has a significantly greater affinity for unstable transition states, thereby reducing the energy barrier during the reaction. Owing to their enzymatic specificity, catalyzed reactions often yield transition state structures with distinct characteristics. This allows the design of a stable compound capable of mimicking the spatial structure, hydrophobicity, and electron distribution of an enzyme-catalyzed reaction, with the aim of obtaining an enzyme inhibitor with both high affinity and selectivity.^372^

A crucial aspect in combating persistent HIV-1 infections involves the development of antivirals that inhibit virus-encoded aspartic proteases. This key enzyme represents a triple threat target, as its inhibition disrupts at least three steps in the viral maturation process. The intermolecular cooperativity manifests as a cooperative dose‒response for inhibition, where the apparent potency increases with greater levels of inhibition. Additionally, the pleiotropic effects of protease inhibition impact viral entry, RT, and post-RT processes. These compounds also exhibit potent activity as transition state analogs, offering potential for further optimization to prevent the virus from developing resistance in the context of monotherapy.^373^

Extensive research endeavors have yielded high-affinity inhibitors, with most analogs featuring a transition state characteristic—a sp^3^ center—where the substrate carbonyl reaction center is reduced to a secondary alcohol that occupies the catalytic site. The sensitive peptide-bound sp^2^ carbonyl group in the transition state is converted into a sp^3^ secondary alcohol within the gem-diol intermediate, ultimately leading to peptide bond loss during this transitional phase (Fig. 9e).^372,374^ By maintaining the structural integrity of the transition state and subsequently optimizing other substituents, Arun K. Ghosh et al. synthesized compounds (**10**) that exhibit exceptional binding affinity.^375^

### Design of antiviral drugs on the basis of the topological structure

#### Molecular tweezers

Molecular tweezers are compounds that mimic the function of tweezers at the molecular level. They consist of two interacting sites and a spacer connecting the two sites. Molecular tweezers have the ability to inhibit protein aggregation or enzyme activity by binding to specific amino acid residues. This property makes them potentially useful for applications in controlled drug delivery. Additionally, they have potential for applications in treating diseases such as Alzheimer’s disease and other protein misfolding disorders.^376,377^

**Fig. 10:**
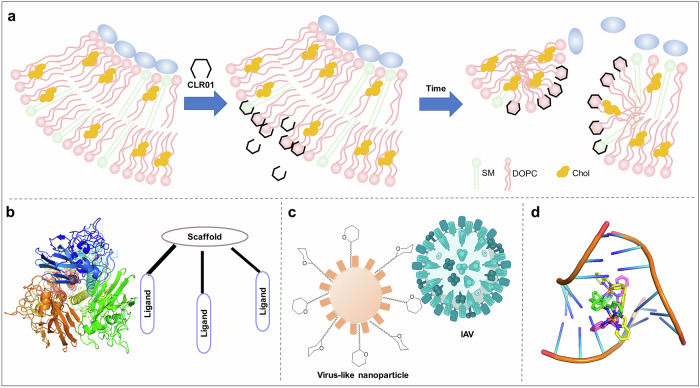
Topological structure-guided antiviral drug design. **a** Schematic representation of virus disruption by molecular tweezers. **b** Tripodal ligand design for chelation-type inhibition of the IAV HA protein. **c** Proposed binding patterns between virus-like nanoparticles and influenza virus particles. **d** Crystal structure view of supramolecular ligands bound in RNA (PDB entry: 4JIY), with biological assembly generated by PISA software

Broad-spectrum antiviral drugs are a potent defense against virulent pathogens. Jan Münch et al. synthesized supramolecular ligands specifically targeting lysine and arginine residues, including CLR01, that can dismantle enveloped viruses such as HIV, Ebola, and Zika while reshaping amyloid fibrils in semen to facilitate viral infection. An investigation of the tweezer CLR01 revealed a novel mechanism underlying its antiviral activity. Notably, CLR01 forms enclosed inclusion complexes with the lipid head groups of viral membranes, thereby altering lipid orientation and augmenting surface tension (Fig. 10a). This process leads to destruction of the viral envelope, reducing infectivity without compromising the integrity of the viral membrane.^378^ The incorporation of hexyl or heptyl ester arms into CLR01 enhances its antiviral activity while maintaining low toxicity levels.^379^

#### Multivalent ligands

Multivalency has been extensively explored as a strategy to enhance binding and inhibitory effects in protein‒carbohydrate interactions by bridging multiple binding sites or inhibiting pathogen attachment through mechanisms such as chelation and statistical rebinding. IAV, which is responsible for causing the flu, poses a significant threat to human health. Among the two envelope glycoproteins of IAV, hemagglutinin (HA) specifically binds to sialylated glycans, facilitating viral adhesion to the surface of infected tissues.^380^

The multivalency effects encompass the simultaneous binding of glycans to multiple binding sites on each HA trimer present on the IAV surface, as well as the concurrent interaction between cell surface glycans and multiple HA protein trimers on the virus envelope. On the basis of the structural characteristics of HA, Roland J. Pieters et al. synthesized multivalent carbohydrate-based ligands.^380^ By conjugating sialylated units to bivalent and trivalent scaffolds, the resulting inhibitors exhibited greatly enhanced inhibition (up to 428-fold) in multiple trials (Fig. 10b).

#### Topology-matching design

Topology-matching design is an innovative strategy in drug design that aims to create inhibitors with a matching topology by simulating the surface features of a virus or other target. The core concept of this approach is to neutralize or inhibit the virus by leveraging the interaction between the surface topology of the drug and the surface protein of the virus.

From a topological perspective, IAV virions are nanosized particles measuring approximately 100 nm in diameter and featuring a pointed surface morphology.^381^ For inhibitor development, particularly for nanoparticle-based inhibitors (nanoinhibitors), achieving strong binding affinity with virions to counteract virus/cell interactions necessitates precise matching of both size and topology. Employing the principle of topology-matching design, Rainer Haag et al. synthesized a virus-like nanoparticle decorated with nanospikes by using linear polyglycerol-sialyllactose and LPG-Zanamivir, as depicted in Fig. 10c. This nanoinhibitor has a nanotopology similar to that of IAV particles and has heteromultivalent inhibitory effects on hemagglutinin and NA. Furthermore, these reverse-designed nanoinhibitors significantly reduce viral replication by six orders of magnitude.^382^

#### Supramolecular drugs

Supramolecular drugs are composed of two or more molecules that are formed through noncovalent bonds, such as hydrogen bonds, hydrophobic interactions, van der Waals forces, and π‒π stacking. These collections of molecules can exhibit chemical, physical, and biological properties that differ from those of a single molecule. Supramolecular drugs have the potential to inhibit key steps in the virus’s life cycle, including entry, replication, and assembly. They can be specifically designed to bind to the active sites of viral proteases or other essential enzymes to effectively inhibit viral replication.^383^

The untranslated regions (UTRs) of the viral genome encompass a diverse array of conserved yet dynamic structures that are crucial for viral RNA replication, thereby presenting opportunities for the development of broad-spectrum antiviral therapeutics.^384^ In pursuit of UTR-targeting drugs, Michael J. Hannon et al. examined the potential of nanosized metallo-supramolecular cylinders as effective RNA-binding agents (Fig. 10d). The nanosize agents are larger than traditional small molecules and feature a broad aromatic surface stacked with RNA bases and cationic charges, ensuring robust binding affinity and optimal conformation for the RNA cavity. These unique agents demonstrate exceptional ability to traverse RNA junctions, selectively binding to a 3-base bulge as well as the central cross-4-way junction located in stem loop 5 of the 5’ UTR of the SARS-CoV-2 genome. Moreover, these specialized cylinders potently inhibit the replication of viruses, highlighting their promising potential as novel antiviral agents.^385^

## Drug design for novel mechanisms of action

### Multi-specific approaches

In contrast to traditional drugs, the multispecific binding strategy enhances classical rational drug design by incorporating two additional effects: first, it enables the localization of the target to a specific effector within the cell, thereby facilitating regulation of the target (e.g., PROTAC); second, it allows drug action at a specific site primarily aimed at reducing adverse drug reactions (e.g., antibody‒drug conjugates). Multiple specific binding drugs have the potential to enhance efficacy, but their development and clinical management may require more sophisticated strategies.^386^ While enhancing efficacy, multispecific binding drugs are being refined to minimize cytotoxicity and adverse effects. This involves enhanced drug design, precise dosage control, and thorough patient monitoring with appropriate supportive care. As the mechanisms of action of these drugs become better understood and new drug development continues to advance, more treatment options with reduced toxicity risks are anticipated.^387^

Both multispecific drugs and targeted drugs present advantages and challenges in terms of time and cost, which depend on the type of drug, development strategy, technology platform, and target disease. The development of multispecific drugs may initially require more time and investment because of their complex design and optimization. However, once platform technology is established, subsequent drug development may accelerate. On the other hand, targeted drugs may require less time and cost in the initial stage of development but could necessitate more late-stage investment to address resistance and side effects. Overall, the efficiency of both multispecific binding and targeted drugs in terms of time and cost depends on various factors, including the type of drug, development strategy, technology platform, and target disease. With advancements in biotechnology, we anticipate that research and development of these drugs will become more efficient and economical.

#### PROTAC

**Fig. 11:**
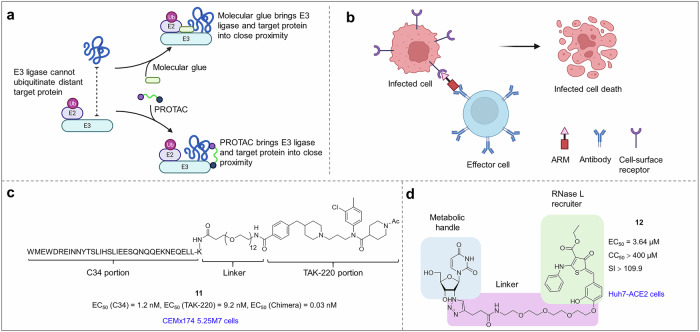
Antiviral drugs using a multispecific binding strategy. **a** Mechanistic overview of PROTACs and molecular glue-mediated protein degradation. **b** Action mode of the ARMs. **c**, **d** Chemical structures and antiviral activities of compounds **11**^407^ and **12**.^411^

The PROTAC molecule is a heterobifunctional entity comprising three components: a ligand for the protein of interest (POI), an E3 ubiquitin ligase, and a linker (Fig. 11a).^387^ Compared with the occupancy-driven inhibitors commonly used, degrading molecules exhibit significantly lower target affinity, rendering them less susceptible to point mutations. This implies that degrading molecules may present resistance profiles distinct from those of conventional enzyme inhibitors.^24,388–390^

HCV, the primary etiological agent of liver disease, such as chronic hepatitis, cirrhosis, and hepatocellular carcinoma, persistently infects an estimated 71 million individuals worldwide.^391^ Telaprevir, a first-generation peptide protease inhibitor, was approved for HCV treatment in 2011. However, concerns over resistance and the emergence of alternative potent direct-acting antivirals led to the withdrawal of telaprevir from the market.^392,393^ TPD as an antiviral strategy is established by utilizing telaprevir as a ligand that binds to the protease. The high-resolution structures of telaprevir and the protease serve as a foundation for designing appropriate attachment sites for the E3-recruiting moiety.^394^

Priscilla L. Yang et al. designed a linker chain in the solvent-exposed region where the pyrazine ring is located, and extensive investigations into various chemical binders led to the acquisition of the PROTAC molecule.^391^ The PROTAC molecule has inhibitory and inducible effects on the degradation of the HCV NS3/4 A protease, thereby contributing to its antiviral activity. This novel class of antiviral agents has potential for overcoming viral variants that develop resistance to conventional enzyme inhibitors such as telaprevir. TPD represents a promising approach for developing antiviral drugs with enhanced resistance profiles.^391^

PROTAC strategies have also been employed in studies targeting influenza, HIV-1 and SARS-CoV-2.^87,395–399^ Although PROTACs are more powerful than other small-molecule inhibitors and completely deplete the target proteins in cells, they may cause toxicity in host cells and may impair normal cellular function.^400^ Some of the POIs engaged by PROTACs can have both enzymatic functions and other scaffold functions that may be important for normal cell proliferation. Therefore, their complete elimination may be harmful to cells.

#### Molecular glue

The field of TPD has undergone rapid advancements in recent years. Numerous PROTAC and molecular glue projects worldwide have progressed into clinical practice, with several leading initiatives regularly disclosing clinical research data. Additionally, novel technologies continue to emerge, including lysosome-targeting chimeras (LYTAC), autophagosome-tethering compounds (ATTEC), asialoglycoprotein receptor-targeting chimeras (ATAC), and autophagy-targeting chimera (AUTOTAC).^401^

Molecular glues facilitate dimerization or colocalization of two proteins through the formation of ternary complexes, thereby exerting regulatory control over diverse biological processes, including transcription, chromatin remodeling, protein folding, subcellular localization, and degradation (Fig. 11a).^127^ Molecular glue degraders promote interactions between the ubiquitin ligase and the POI, resulting in ubiquitination and subsequent degradation of the POI. Unlike molecular glues, PROTACs are heterofunctional degraders that simultaneously interact with both the E3 ligase and the POI. Conversely, molecular glue degraders can engage solely with either ligases (which are more prevalent) or POI, accelerating or stabilizing their interactions. Furthermore, compared with PROTACs, molecular glues lack linkers, rendering them favorable drug-like properties, including lower molecular weights, fewer hydrogen bond donors, and fewer rotatable bonds. These characteristics contribute to the enhancement of oral bioavailability. However, designing molecular glues remains a challenging task despite the emergence of rational design strategies.

To design molecular glues with precision, a transposable chemical handle capable of converting protein-binding ligands into molecular degraders was identified. Daniel K. Nomura et al. identified and optimized covalent handles for this purpose. By incorporating this covalent handle onto a diverse set of protein-binding ligands, degradation of various proteins, such as CDK4, BRD4, BCR-ABL, c-ABL, PDE5, AR, AR-V7, BTK, LRRK2, HDAC1/3 and SMARCA2/4, has been achieved.^402^

#### Antibody recruiting molecule (ARM)

Antibody-recruiting molecules can recruit endogenous antibodies to the surface of specific cells, thereby activating the immune system for recognition and elimination of infected cells through an immune-mediated mechanism. This class of bispecific small molecules comprises three domains: an antibody-binding terminus (ABT), a cell surface-binding terminus (CBT), and a linker.^387^

The zanamivir-dinitrophenyl (DNP) conjugate was designed by combining the NA inhibitor zanamivir with the highly immunogenic hapten DNP. Philip S. Low et al. utilized SPR assays and confocal microscopy to demonstrate that conjugates (Fig. 11b) exhibit robust binding affinity toward NA while also showing concentration-dependent recruitment of anti-DNP antibodies. This conjugate effectively inhibits both influenza A and B virus NA activity, simultaneously mobilizing the immune system to recognize and eliminate virus-infected cells.^403^

#### Antibody‒drug conjugates

Antibody‒drug conjugates represent a novel class of targeted therapeutics characterized by large molecular carriers comprising antibodies, cytotoxic small molecules, and linking chains.^404^ Leveraging the specificity of antibodies for antigens on the surface of target cells, antibody‒drug conjugates efficiently deliver cytotoxic drugs into the intracellular space of these cells, inducing their destruction through various immune mechanisms within the body. Both antibody‒drug conjugates and antibodies act in a covalent binding manner to eliminate target cells.

An effective binding site was identified for the CCR5 inhibitor maraviroc. By conjugating it to mAb 38C2 through a linker, Carlos F. Barbas et al. designed an antibody‒drug conjugate. Consequently, this study successfully identified viable binding sites for maraviroc and applied a multipiece binding strategy for antibody‒drug coupling to exert antiviral effects.^405^

#### Short-peptide-based inhibitors

The development of short peptide inhibitors to prevent HIV-1 entry into host cells has achieved limited success. A multitarget-directed ligand strategy was employed to generate a series of short peptide inhibitors for virus entry. They synergistically integrate the pharmacological activity of a peptide fusion inhibitor. which disrupts the assembly of the gp41 glycoprotein hexameric coiled-coil structure and a small-molecule CCR5 antagonist that inhibits the interaction between the virus and its coreceptor.^406^ Shibo Jiang et al. developed CP12TAK (**11**, Fig. 11c), which exhibited potent antiviral activity, demonstrating approximately 40- and 306-fold greater efficacy than its parent inhibitors, C34 and TAK-220, respectively. This approach can be further extended for the development of therapeutic interventions against other enveloped virus infections.^407^

#### RNA degradation strategies

Owing to its intricate structure, RNA has a diverse array of cellular functions, including the regulation of gene expression, protein translation, and the cellular response to stimuli. Small molecules can selectively bind to functional structures within intracellular RNA and modulate their activity; for example, C5 has a strong affinity for the revised attenuator hairpin structure, with a dissociation constant (*K*_d_) of 11 nM. This compound effectively stabilizes the folded state of the hairpin and hinders frameshifting within cellular environments.^408^ However, mere binding interactions often prove inadequate, resulting in diminished or negligible biological efficacy. To overcome this challenge, heterobifunctional compounds have been designed that can covalently engage with RNA targets, thereby modifying their sequence or inducing cleavage.^409^

Nature has developed intricate mechanisms for RNA degradation, some of which can be harnessed for therapeutic purposes. The *β*-coronavirus pseudoknot is an RNA structure found in the genome of SARS-CoV-2. By linking a pseudoknot binder, a linker, and an imidazole degrader, Sigitas Mikutis et al. designed and synthesized a series of novel molecules that could effectively degrade their targets in vitro, *in cellulo*, and in vivo infection models. This discovery opens the possibility of degrading any disease-associated RNA species, thereby greatly expanding potential drug interventions for various diseases.^410^

Furthermore, a nucleoside tailoring strategy offers a similar approach to degrade RNA, addressing the challenges of RNA recognition by seamlessly integrating RNase L recruiters into nucleosides. Notably, Xiang Zhou et al. designed and synthesized compound **12** (Fig. 11d), which has significant efficacy in inhibiting SARS-CoV-2 replication in human cells. The validation of this nucleoside tailoring approach through in vivo experiments using hamster models further strengthens its potential.^411^

### Virus-targeted structural proteins

In addition to the NSPs, the structural proteins of viruses are also a major focus of research. Coronavirus virions encode four structural proteins that form the virion. Drug development targeting these structures has been fruitful. Among HBV CA inhibitors, several drugs have entered phase II clinical trials, with GLS4 being the first nucleocapsid inhibitor to enter phase III clinical trials globally.^412^

#### Targeted viral membrane

Lipid bilayer-enveloped viral membrane-rafted proteins are considered broad-spectrum antiviral targets, and compounds affecting them have been reported. Viroporins, viral-encoded ion channels, facilitate the migration of protons or other cations through the viral membrane or cellular vesicles, controlling acidification or deacidification within the compartments. Zhiqiang Lin et al. constructed a library of gradient pH-sensitive (GPS) polymeric nanoprobes, and the transition of the cell membrane pH from pH 6.8–7.1 (uninfected) to pH 6.5–6.8 (virus-infected) was measured. At a pH of 6.8, the GPS polymer selectively binds to the viral envelope or infected cell membrane and can even completely disrupt the viral envelope structure. Consequently, treatment with GPS6.8 inhibits various viruses, including SARS-CoV-2, by inducing cleavage of the viral envelope. In a mouse model of viral infection, supplementation with GPS6.8 reduced viral titers and ameliorated inflammatory damage.^413^

#### Targeted viral RNA

Several RNA viruses, including SARS-CoV-2, harbor unique RNA structures that enable programmed ribosomal frame-shifting (PRF) for the precise regulation of viral gene expression.^414^ Through high-throughput compound screening, Junjie U. Guo et al. reported that a potent PRF inhibitor (merafloxacin) robustly impeded viral replication in cultured cells. Interestingly, merafloxacin has potential against not only SARS-CoV-2 but also other coronaviruses that employ RNA structures for frame shifting. These findings suggest that targeting PRF represents a plausible broad-spectrum antiviral strategy against different coronaviruses.^415^

The 5’ UTR refers to the noncoding region upstream of the coding sequence (CDS) in mature mRNA, which is not translated into protein. Targeting conserved structural elements within the viral 5’-end as a strategy for inhibiting OC43 and SARS-CoV-2 replication represents a promising approach in drug design. NMR-assisted structural studies have revealed specific interactions between amiloride-based small molecules and stem rings containing bulge structures, demonstrating its antiviral activity in SARS-CoV-2-infected cells.^416^

G-quadruplexes (G4s) are crucial noncanonical nucleic acid structures that play diverse biological roles. RG-1, located within the coding sequence region of the N phosphorylated protein, has been demonstrated to form a stable RNA G4 structure in vivo. Xiaogang Qu et al. demonstrated that the pyridostatin derivative (4-(2-aminoethoxy)-*N*^2^,*N*^6^-bis(4-(2-(pyrrolidin-1-yl)ethoxy)quinolin-2-yl)pyridine-2,6-dicarboxamide) effectively stabilizes RG-1 G4 and reduces viral N protein levels by inhibiting its translation.^417,418^

#### Virus entry inhibitor

The RBD of the SARS-CoV-2 S protein is endocytosed via a pH-dependent pathway. Endosomal acidification inhibitors, such as bafilomycin A1 and NH_4_Cl, impede this pathway, resulting in decreased uptake of the RBD and prevention of spike-pseudovirus infection. Furthermore, Satyajit Mayor et al. identified niclosamide through a screening assay as a potent inhibitor of virus entry, effectively neutralizing the endosomal pH and blocking the uptake of viral particles via pH-dependent endocytosis pathways. This discovery highlights its broad applicability in disrupting viral infections that rely on such mechanisms for host cell entry.^419^

#### LLPS

LLPS is a novel mechanism for the generation of cell compartments and condensates, known as membraneless organelles (MLOs).^420^ MLOs perform crucial functions by spatially confining specific proteins and nucleic acids to distinct subregions, enabling simultaneous or mutually exclusive biochemical reactions.^421^ The formation of condensates serves as the fundamental basis for the organization and compartmentalization of intracellular biochemical processes, thereby facilitating viral infection.^422,423^ Furthermore, LLPS plays a pivotal role in various stages of the viral life cycle, including viral entry and uncoating,^424^ synthesis and replication of the viral genome,^425,426^ virion assembly,^427^ budding,^428^ and evasion and sensing mechanisms against innate immune responses.^429–432^ Thus, LLPS holds substantial potential in drug discovery, with wide-ranging implications.

Advancements in the field of biomolecular condensates have presented new opportunities for nonpatentable drug targets. Numerous nontargetable drug targets, such as transcription factors, contribute to the formation of biomolecular condensates through disordered protein regions. Modulating the phase separation regulated by these disordered regions holds immense potential for influencing the functionality of these “nontargetable” targets at an unprecedented level.^433^

In SARS-CoV-2, the combination of a purified N protein with cellular or viral genomic RNA leads to condensate formation. Phase separation of the N protein promotes viral genome packaging, virion assembly, replication transcription complex formation, and virus-induced inflammation. Antiviral drugs that increase the conversion of viral proteins from the liquid phase to the solid phase or disrupt the LLPS of viral proteins can markedly affect viral replication.^434^ LLPS modulators can be utilized to manipulate these condensates and are classified into condensate inhibitors and pro-condensation modulators on the basis of their effects.^429^

Jianyang Zeng et al. reported that the PARP inhibitor CVL218 (*K*_D_ = 4.7 μmol/L) has significant binding affinity for the N protein. In the presence of CVL218, the size of the N-RNA condensates increased. Mechanistically, CVL218 reduces the local density of N-NSP12 condensates and enhances their permeability, allowing other small-molecule drugs to enter these condensates. Consequently, combining CVL218 with remdesivir can augment the therapeutic efficacy against SARS-CoV-2.^435^

Tao Li et al. demonstrated that (–)-gallocatechin gallate (GCG), a polyphenol extracted from green tea, effectively inhibits the LLPS of the N protein at a concentration of 12.5 μM. GCG directly binds to the N protein and hinders its RNA-binding ability. Notably, one of the isomers of GCG, epigallocatechin gallate, has significantly diminished efficacy in preventing N-RNA condensation, highlighting the critical role of compound chirality in LLPS.^429^

Currently, most LLPS modulators are identified primarily by phenotypic screening via FDA-approved libraries. Exploring larger, more structurally diverse libraries should be the next step. Although developing LLPS as a selective pharmacological modulator for antiviral strategies has been challenging, LLPS holds promise for providing new treatment options for currently incurable viral diseases in the near future.

### Regulating viruses with organelle-targeted drugs

Accurate targeting poses a fundamental challenge for effective treatment, and the prospect of focusing on specific organelles offers potential in enhancing therapeutic efficacy and overcoming cellular resistance, thereby bearing significant clinical implications. As our understanding of disease progression deepens, particularly with respect to the intricate interplay of biomaterials and their behavior within living cells, an increasing number of drugs targeting various stages of the virus life cycle will be developed.

Organelle dynamics encompass a range of processes, including organelle biogenesis, movement, fusion, fission, and degradation. The replication and dissemination of RNA-coated viruses rely heavily on hijacking host cell organelles. Viral entry into host cells involves various pathways that involve different organelles, such as the plasma membrane, endosome, lysosome or Golgi apparatus. Additionally, viruses exploit cytoskeletal elements and motor proteins for intracellular transport toward replication sites. Retrograde transport from the plasma membrane to the nucleus or other organelles is mediated by microtubules and associated proteins in certain cases. Consequently, targeting subcellular homeostasis has emerged as a promising strategy for antiviral interventions.

#### Selective inhibitor of nuclear export

The nuclear export protein XPO1 plays a direct role in the transportation of SARS-CoV proteins, including ORF3b and ORF9b. Inhibition of XPO1 induces pathways with anti-inflammatory, antiviral, and antioxidant effects. Selinexor is an FDA-approved inhibitor that targets XPO1. Yosef Landesman et al. demonstrated that selinexor treatment significantly reduced the viral load in the lungs and protected against tissue damage in both the turbinate bone and lungs.^436^ Selinexor effectively impedes the nuclear‒cytoplasmic transport of the SARS-CoV-2 nuclear protein, thereby exerting inhibitory effects on viral proliferation. Additionally, selinexor has the potential to mitigate cytokine storms associated with COVID-19 by suppressing proinflammatory cytokine release while promoting the secretion of anti-inflammatory cytokines.^437^

#### Targeted exosome

Exosomes are extracellular vesicles that adhere to the plasma membrane of various cell types and can be released by almost all cell types.^438^ Exosomes are formed through the inward budding of the endosomal membrane, resulting in the pinching off and release of intraluminal vesicles (ILVs) inside endosomes. These ILVs then become known as multivesicular bodies (MVBs).^439^ HBV exploits MVBs as a platform for their budding and subsequent release from host cells.^440^ Therefore, developing medications that affect exosomes has the potential to inhibit viral replication.

The Ebola virus modulates exosome biogenesis in infected cells through its viral protein 40 (VP40). VP40 is packaged within exosomes, which impair and eventually dismantle the T-cell and myeloid components of the immune system, allowing robust replication of the virus in immunocompromised hosts. Fatah Kashanchi et al. reported that oxytetracycline modulates the levels of exosomes containing VP40, thus preventing damage to the adaptive immune system and increasing survival in immunocompromised hosts.^441^

Certain drugs alter the number and composition of exosomes, reducing their viral characteristics. However, there is a worrying shortage of drugs that specifically regulate exosomes to combat viral infections.

#### Targeted lysosomes

Antiviral activity can be achieved by regulating lysosomal function or promoting lysosomal degradation.^442^ *β*-Coronaviruses, including SARS-CoV-2, utilize lysosomal trafficking for egress. The nonlytic release of *β*-coronavirus triggers lysosomal deacidification, inactivation of lysosomal degrading enzymes, and interruption of antigen presentation pathways. This unconventional export process provides insights into potential therapeutic modalities. Nihal Altan-Bonnet et al. reported that the small ARF-like GTPase Arl8b regulates this mechanism and can be inhibited by the Rab7 GTPase competitive inhibitor CID1067700.

Numerous viral pathogens have evolved intricate mechanisms to evade or manipulate the endolysosomal pathway, exemplified by their ability to trigger proteasome-mediated degradation of the key transcriptional regulator EB (TFEB), which is responsible for lysosomal biogenesis. The stability of the TFEB protein is tightly regulated by the E3 ubiquitin ligase subunit DCAF7 and p21-activated kinase 2 (PAK2). Inhibition of either DCAF7 or PAK2 effectively prevents virus-induced TFEB degradation, thereby mitigating virus-mediated cytopathic effects. Notably, Bill Chen et al. discovered that compounds such as BC18630 (*N*-(1*H*-indol-5-yl)-2-(pyridin-4-yl)quinazolin-4-amine hydrochloride), which inhibits DCAF7 activity, exhibit broad-spectrum antiviral properties, including alleviation of SARS-CoV-2 infection in vivo. These findings substantiate that the modulation of lysosomal function represents a promising therapeutic strategy against coronavirus infections.^443^

Moreover, numerous viruses exploit the host’s internal lysosomal networks to trigger infection.^444^ The RBD of the SARS-CoV-2 S protein is internalized through the pH-dependent CLIC/GEEC endocytosis pathway, exemplifying a mechanism for viral entry.^419^ In addition, chloroquine, which modulates endosomal pH, can impede the internalization process and exhibit broader efficacy in eradicating viral pathogens that exploit the pH-dependent endocytic pathway for host cell entry.

### Targeted activator of cell kill (TACK) molecules

Antiretroviral therapy (ART) is employed to suppress the replication of HIV-1. However, owing to viral integration into the host genome and the subsequent formation of stable reservoirs, ART does not provide a complete cure for HIV. Accordingly, reducing these reservoirs has emerged as an imperative strategy pursued by researchers in their quest for an HIV cure.^445^

Certain NNRTIs that target HIV-1 exhibit selective cytotoxicity under experimental conditions, preferentially eliminating HIV-1-infected cells. Nevertheless, this effect necessitates drug concentrations surpassing standard dosages. Through exploration of this side effect, a bifunctional compound has been discovered that effectively eradicates HIV-1-infected cells at clinically acceptable drug concentrations. This compound is referred to as TACK, a cell killer molecule. Antonella Converso et al. designed the TACK molecule (5-(difluoromethyl)-3-((1-(5-fluoro-2-oxo-1,2-dihydropyridin-3-yl)methyl)-6-oxo-4-(1,1,2,2-tetrafluoroethyl)-1,6-dihydropyrimidin-5-yl)-2-methylbenzonitrile), which can bind to the RT-p66 domain, a component of the HIV Gag-Pol protein, acting as an isomeric regulator that promotes dimerization of the protein. This leads to premature activation of viral proteases, resulting in the death of HIV-1-positive cells. TACK molecules exhibit potent antiviral activity and selectively eliminate CD4^+^ T cells infected with HIV-1, a subset of white blood cells. This finding supports an immune system-independent clearance strategy, motivating scientists to eradicate HIV-1-infected cells without relying on immune system assistance through the use of TACK molecules.^445^

## Property-based drug design

The design of drugs on the basis of their properties involves the meticulous consideration and regulation of molecular and physicochemical attributes to optimize the pharmacological and safety profiles of drug molecules, encompassing their pharmacodynamic, pharmacokinetic, and toxicokinetic properties.^446^

The druggability properties are typically determined by the drug’s requirements, necessitating that the lead compound be chemically facile to synthesize and possess desirable ADME (absorption, distribution, metabolism, excretion) characteristics. Furthermore, these druggability properties encompass both the physical and chemical attributes (such as solubility, lipophilicity, dissociation constant, and stability) as well as the biological properties of the compounds. These crucial features play a pivotal role in evaluating potential compound failures and mitigating development risks.

### Solubility optimization

The solubility of drugs is a critical factor that significantly impacts their efficacy. It directly influences the ADME of drugs within the body. The solubility of a drug significantly impacts its bioavailability, dosage form selection, formulation design, drug release and absorption, as well as the required dose and frequency of administration.

During the HIV-1 life cycle, RT plays a pivotal role in catalyzing the conversion of single-stranded RNA into double-stranded DNA through reverse transcription. Given its critical involvement, RT has emerged as an exceptionally promising target for the development of therapeutic agents against HIV-1.^447^

Diarylpyrimidine (DAPY) derivatives represent a promising class of second-generation nonnucleoside RT inhibitors. Among them, etravirine and rilpivirine have gained approval from the U.S. FDA. However, to enhance RT binding affinity, lipophilic aromatic rings were introduced, which resulted in poor water solubility, possibly due to intermolecular π‒π stacking interactions. Therefore, there is an imperative need for the development of novel DAPYs with improved potency and solubility.^448^

**Fig. 12:**
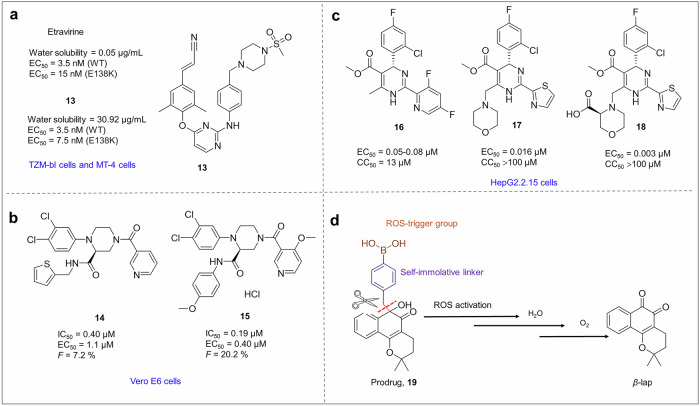
Representative examples of property-based drug design. **a** Compound optimization to improve water solubility.^449^ **b** The enzyme-inhibitory potency, antiviral activity, and pharmacokinetic properties of the compound were improved by salt formation.^451^ **c** Cytotoxicity was reduced by modifying the structure of the compounds.^452^ **d** A C‒C bond-cleaving strategy was applied to design *β*-lap prodrugs

Our group employed a scaffold hopping strategy to optimize the structure of DAPY (**13**, Fig. 12a). This strategic modification not only resulted in significantly improved drug resistance profiles but also enhanced drug solubility and bioavailability. Furthermore, these structurally modified compounds exhibited favorable pharmacokinetic properties, including oral availability and extended half-life, highlighting their potential for further development.^447–449^

### Salt formation

Salt formation is an effective strategy for enhancing the physical and chemical properties of drugs, thereby improving their druggability. Among these, hydrochloric acid, sulfuric acid, and hydrobromic acid are frequently utilized for salt formation with organic bases. The commonly employed bases for forming salts with organic acids include sodium hydroxide, calcium hydroxide, and potassium hydroxide.^450^

By incorporating a methoxy group and optimizing the substituents, our research team successfully identified potent nonpeptide M^pro^ inhibitors. Compound **14** (Fig. 12b) was subjected to salt formation treatment, resulting in the synthesis of compound **15**, which has enhanced activity and improved pharmacokinetics. Notably, compound **15** exhibited robust antiviral efficacy against various variants as well as HCoV-OC43 and HCoV-229E, highlighting its potential for broad-spectrum anti-coronavirus activity.^451^

### Reducing cytotoxicity

The cytotoxicity of compounds refers to their deleterious effects on cells, encompassing a range of adverse consequences, such as cellular death, DNA damage, and alterations in cell morphology. Cytotoxicity holds paramount importance in drug development and environmental monitoring, serving as a pivotal factor for assessing the safety profile and potential risks associated with these compounds. To reduce the cytotoxicity of compounds, scientists typically explore multiple aspects, including compound structure, administration methods, and drug combinations. By optimizing the structure of a compound, its cellular toxicity can be reduced.^449^

The heteroaryldihydropyrimidine (HAP) compound, represented by compound **16**, is a unique inhibitor of HBV CA assembly (Fig. 12c). It effectively disrupts the normal assembly of core proteins and leads to the formation of abnormal CAs. Previous studies have reported the hepatotoxicity of compound 16 in rats at high doses. Subsequently, second-generation analogs with improved potency were developed by introducing the C6 morpholinyl group, including compound **17** from the 4-H HAP series of CA inhibitors. However, compound **17** exhibited high liver microsome clearance. To address this issue, Wei Zhu et al. introduced polar substituents, including hydroxyl, sulfanilamide, carboxyamide, and carboxylic groups, to reduce the alkalinity and lipophilicity of second-generation 4-H HAP analogs. This led to the discovery of more effective drug analogs. The introduction of a carboxyl group at the *α*-position of C6-morpholine was well tolerated and resulted in analog **18** being five times more potent than compound **17**, with a mean EC_50_ value of 3 nM in HepG2.2.15 cells.

The incorporation of an ionizable carboxyl group significantly diminished the lipophilicity of analog **18** (log P = 0.3) while concurrently increasing its aqueous solubility (LYSA > 666 μg/mL); moreover, this compound presented a CC_50_ value exceeding 100 μM and significantly improved the preliminary ADME and in vitro safety assessment.^452^

### Targeted drug delivery

Targeted drug delivery is a strategic approach in drug administration that aims to deliver therapeutic agents precisely to specific tissues or sites, thereby enhancing therapeutic efficacy while minimizing adverse reactions. By selectively utilizing molecules such as antibodies, ligands, or peptides, it becomes possible to bind them specifically to receptors or markers on the surface of target cells or tissues. These targeted molecules possess the ability to recognize and attach themselves exclusively to the intended region, facilitating precise drug delivery.^453^

Compared with that in other tissues, the expression of the asialoglycoprotein receptor (ASGPR) on the liver cell membrane is significantly greater. ASGPR exhibits specific recognition of structures containing galactose or acetylgalactosamine, thereby offering potential for liver-targeted delivery and reduced systemic toxicity when galactose and acetylgalactosamine-modified drug-carrying nanoparticles or biomacromolecules are utilized.^454^

GalNAc binds to the ASGPR expressed on hepatocytes, facilitating rapid endocytosis of GalNAc conjugates following intravenous or subcutaneous administration. Notably, subcutaneous injections of GalNAc conjugates were well tolerated in mice, rats, and nonhuman primates and resulted in a prolonged gene silencing period of 140 days in humans.^455^

### Prodrug strategy

Prodrugs are a class of pharmaceutical agents that exhibit pharmacological activity subsequent to undergoing a series of biotransformations within the human body. The crux of this technology lies in employing specific chemical modifications, thereby maintaining drug inactivity or low activity until it reaches the intended target site, with the ultimate aim of mitigating adverse effects and enhancing therapeutic efficacy.^456^

In the design of antiviral drugs, prodrug technology is highly important because of the high variability and replication ability of viruses. Traditional antiviral drugs often face challenges in completely eradicating the virus and are prone to resistance development. Prodrug technology enables the gradual release of active substances within the body, thereby ensuring the sustained efficacy of the drug and effectively inhibiting viral replication and infection.^457^

Currently, prodrug technology has been successfully employed with a diverse range of antiviral drugs, including those targeting HIV, SARS-CoV-2, and hepatitis viruses (such as HBV and HCV). These drugs undergo enzymatic conversion within the body to generate active compounds that exhibit increased viral specificity while minimizing off-target effects on normal cells. Furthermore, prodrug technology is continuously evolving and advancing. Scientists are actively exploring novel strategies for prodrug design to increase the efficacy and safety of antiviral medications. For example, by modifying the chemical structure of the prodrug or introducing innovative metabolic pathways, it becomes possible to tailor the drug’s properties according to specific viral infection characteristics, thereby enhancing its broad-spectrum activity and therapeutic effect against various viruses.^458^

#### Protein nucleotide (ProTide) prodrug

ProTide technology represents a prodrug approach for achieving efficient intracellular delivery of nucleoside analogs monophosphate/monophosphonate. The design concept behind ProTide involves the utilization of an aromatic group and an amino acid ester group to mask the hydroxyl moiety of monophosphate/monophosphonate, thereby enabling the resulting prodrug molecules to liberate free nucleoside monophosphate/monophosphonate through intracellular enzymatic catalysis upon cellular internalization, thus facilitating their biological activity. Currently, ProTide technology has extensive applications in drug discovery and design.^456^

Remdesivir, a prodrug form of GS-441524 monophosphate that disrupts viral replication, was initially assessed in a clinical trial in 2014 to combat the Ebola outbreak. Subsequent evaluations conducted in numerous virology laboratories demonstrated the ability of remdesivir to inhibit the replication of coronaviruses, including SARS-CoV-2. Remdesivir can be erroneously incorporated by an RdRp into an elongating RNA strand, thereby impeding viral replication. Within the cell, remdesivir undergoes metabolism to form an alanine metabolite, which is further processed into a monophosphate derivative and ultimately generates an active nucleoside triphosphate derivative.^90^

#### Long alkyl acid chain Prodrug

The limitations of nucleosides primarily arise from their hydrophilic nature, which impedes efficient transport across the lipid bilayer membrane. The prodrug strategies investigated thus far have aimed to mask negative charges by incorporating enzymatically cleavable hydrophobic chemical moieties that facilitate intracellular permeation. Various chemical approaches explored include acyloxyalkyl, alkyloxyalkyl, acylthioethyl, aryl, acyloxybenzyl phosphonate ester, cyclosaligenyl phosphonate ester, phosphonamidate, and tyrosine phosphonate ester derivatives. The incorporation of a lipid moiety enables enhanced cellular uptake and improved efficacy against a wide range of dsDNA viruses.

Cidofovir (CDV) is used against cytomegalovirus and adenovirus infections but has limited clinical utility because of its poor bioavailability and high occurrence of acute nephrotoxicity. Elke Lipka et al. attached a long sulfonyl alkyl chain to one of the phosphono oxygens, resulting in prodrug compounds, which exhibit enhanced aqueous solubility, optimized metabolic stability, increased cellular permeability, and rapid intracellular conversion to the pharmacologically active diphosphate form. Single-dose intravenous and oral pharmacokinetic experiments demonstrated that the prodrug compound maintained significantly higher plasma and target tissue CDV levels than the EC_50_ for up to 24 h.^459^

#### Carbon‒carbon (C‒C) bond-cleaving prodrug

The incorporation of a prodrug strategy is an essential component of contemporary drug design, playing a pivotal role in enhancing the physicochemical properties, efficacy, and safety profiles of pharmaceutical agents. Despite extensive research efforts dedicated to prodrug design over recent decades, current activation strategies predominantly rely on the cleavage of C-N/C-O bonds within conventionally modifiable functional groups such as hydroxyl, amino, and carboxyl moieties. Consequently, there is a pressing need for novel prodrug activation approaches tailored to drugs lacking these conventional modification sites.

Xiaojin Zhang et al. reported a prodrug activation strategy that relies on the cleavage of C‒C bonds, successfully demonstrating the design of *β*-lapachone (*β*-lap) prodrugs.^460^ The designed prodrug of *β*-lap (**19**, Fig. 12d) is rapidly activated upon exposure to a reactive oxygen species (ROS)-specific trigger, leading to the release of *β*-lap. This prodrug exerts potent anticancer effects through NAD(P)H: quinone oxidoreductase 1 (NQO1)-mediated futile redox cycling and demonstrates remarkable selectivity and cytotoxicity toward NQO1-rich cancer cells both in vitro and in vivo.

## Emerging technologies have promoted the field of drug discovery

### Medical chemistry

Pharmaceutical chemistry plays a pivotal role in the exploration and advancement of novel drugs. By delving into the comprehensive study of natural or synthetic compounds, pharmaceutical chemists can unravel novel mechanisms of drug action and identify innovative therapeutic targets. Concurrently, they can also unearth drugs with novel modes of action through the design and synthesis of new compounds. Progress in chemistry has further equipped researchers with an array of tools for drug development.

#### Multicomponent reaction

The multicomponent reaction offers convenience in compound construction, characterized by its simplicity of operation, high resource utilization, and economic efficiency. In the research and development of SARS-CoV M^pro^ inhibitors, a series of target compounds were synthesized via the Ugi reaction to explore the structure‒activity relationships of different subbinding pockets rapidly. This facilitated the discovery of potential inhibitor drugs.^461^

#### Molecular editing

Accurately controlling the selectivity of multisite molecular skeletons is a significant challenge in organic synthesis and drug development. Despite numerous reaction methods that can convert starting compounds into other compounds, chemists still lack a toolkit to modify C‒H bonds alone.

Bicyclic azaarenes are relatively simple organic molecules, and countless existing drugs and medically relevant natural compounds are built from their skeleton. Jin-Quan Yu et al. designed two distinct guide templates and adjusted factors such as distance, geometric structure, and chirality in the template design, thereby achieving precise modular distinction of remote adjacent (C6/C7) sites and similar (C3/C7) sites on bicyclic azaarenes.^462^ In conjunction with previously established methodologies, it is now feasible to achieve precise and selective molecular manipulation of all C‒H bonds within bicyclic azaarenes. This breakthrough encouraged chemists to synthesize an extensive range of chemical compounds that were previously inaccessible, including potential pharmaceutical agents.

#### High-throughput purification

In the field of drug discovery, drug purification plays a pivotal role and has long been regarded as the bottleneck in the design−make−test cycle of novel drugs. However, recent advancements in technologies and systems have seamlessly integrated into high-throughput synthesis workflows, enabling the production of high-purity compounds that can be directly employed for biological screening.^463^ Ross L. Goodyear et al. proposed an integrated preparation system that incorporates submicrogram chemistry through the use of four- or eight-column high-performance liquid chromatography, MS-guided fraction collection, charged aerosol detection for quantitative analysis, and comprehensive integration to streamline the preparation of screening test plates. This system offers enhanced throughput and the ability to efficiently transform virtual composite ideas into tangible composite libraries at a reduced cost while minimizing environmental impact.

#### Medicinal chemist toolbox

**Fig. 13:**
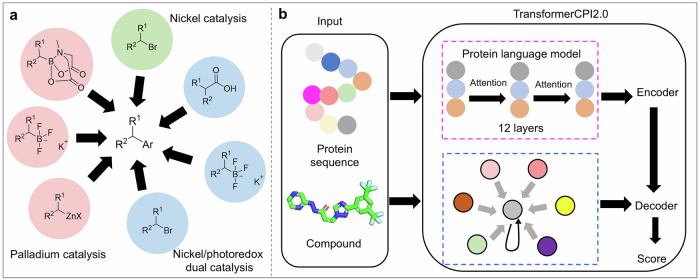
Several emerging technologies have promoted the field of drug discovery. **a** Expanding the medicinal chemical toolbox: representatives of seven C(sp^2^)−C(sp^3^) cross-coupling methods. **b** The computational pipeline of TransformerCPI2.0

To address the issue of drug synthesis, chemists often compare reactions and incorporate newly published methods into their chemical toolbox. In the context of C(sp^2^)−C(sp^3^) cross-couplings, a parallel library synthesis was conducted to evaluate the efficacy of seven different methods (Fig. 13a) in directly introducing various alkyl groups onto the aryl structure of a “drug-like” compound. Compared with alternative approaches, each method exhibited superior coupling capabilities. Upon comprehensive analysis of all methodologies employed, an impressive reaction success rate of 50% was achieved.^464^ The development of several other modular reactions for C‒C bond coupling has also been achieved.^465^ The findings should be utilized as a guiding framework for future synthesis endeavors to evaluate the breadth of reactions and to inspire pharmaceutical chemists in broadening their repertoire of synthetic methodologies.

In the realm of macrocycles, Hong Liu et al. reported a highly efficient and practical methodology for the functionalization and macrocyclization of tryptophan (Trp) and Trp-containing peptides through Pd(II)-catalyzed C–H alkenylation at the C4 position of Trp. These unique macrocycles presented low micro- to submicromolar EC_50_ values, indicating promising anti-SARS-CoV-2 activity.^466^

Given the known limitations of reaction parameters, synthesis design tools, synthetic strategies, and innovative chemistries, there are opportunities for expanding the synthetic toolbox of medicinal chemists. Some emerging technologies, such as continuous flow chemistry, enable reactants to undergo reactions in a microreactor through a continuous flow process. Compared with traditional batch synthesis, continuous flow chemistry offers greater production efficiency and improved reaction control capabilities.^467^ Additionally, enzymatic or cellular reactions occurring within biological systems present advantages such as mild conditions and high selectivity. In contrast, chemical synthesis is characterized by rapid reactions and a broad range of applicability. By integrating biosynthesis with chemical synthesis, it is possible to fully leverage the strengths of both approaches, thereby achieving efficient synthesis of complex pharmaceutical molecules.^468^ Increased collaboration between synthetic and medicinal chemists in both industry and academia is essential to enhance the impact of new methodologies in future drug discovery.^264^

#### Automated synthesis of drug-like molecules

Owing to the high rate of false positives in virtual screening activities, it is necessary to synthesize the expected hits for verification. However, manual synthesis is a time-consuming and labor-intensive process. Large virtual libraries (>7 × 10^12^ members) suitable for automated synthesis via capsule-based technology and commercial building blocks were utilized in an on-demand manner. The library hits identified through virtual screening were automatically prepared for evaluation, resulting in a tenfold improvement in user efficiency compared with manual workflows. To increase the synthesis speed, a simplified relative stereochemical coordination method was also developed. By reducing the size and implementing parallel capsule chemical processing on a 96-well plate equipped with a filter base, the user efficiency was further enhanced by up to 100 times.^469^

### AI-driven drug design

The advancement of computational omics technology has revealed novel potential for drug discovery. Simultaneously, the integration of AI in drug research encompasses genome and metabolome mining, structural characterization of drugs, and prediction of drug targets and biological activity. ML has made remarkable strides in computational drug design by facilitating bioactivity prediction and enabling de novo drug design for specific molecular targets.^470^

AI has made significant contributions to the field of protein structure prediction. Consequently, the 2024 Nobel Prize in Chemistry was partially awarded to Demis Hassabis and John Jumper of DeepMind, an AI company based in London, England, which is owned by Google. In 2020, AlphaFold2,^471^, an AI model capable of predicting nearly all known protein structures, was developed. Since this breakthrough, AlphaFold2 has been utilized by over 2 million users across 190 countries, aiding researchers in understanding antibiotic resistance and other critical areas. In May, Google introduced AlphaFold3,^472^, a revolutionary model that can predict the structure and interactions of all biological molecules. AlphaFold3 demonstrates at least a 50% improvement over existing methods in predicting protein interactions, with some interaction types showing a doubling in accuracy. In addition to proteins, AlphaFold3 will also explore a broader range of biomolecules. This advancement has the potential to transform bioscience research, from the development of biorenewable materials and more resilient crops to the acceleration of drug design and genomics studies.

Several AI techniques have been developed to expedite drug discovery by accurately predicting biosynthetic genes and metabolite structures from sequence or spectral data, respectively. In these more intricate scenarios, ML algorithms have demonstrated substantial advantages over rule-based approaches.^473^

In general, extensive efforts have been dedicated to enhancing the structural characterization of pharmaceuticals through advancements in methodologies, instrumentation, and computational approaches such as quantum chemistry-based theoretical calculations and AI-driven prediction of MS and NMR data structures. AI has demonstrated its potential in predicting molecular formulas from mass spectra, whereas deep neural networks offer a promising avenue for matching mass spectra with compounds stored in molecular databases.^474^ DP4-AI integrates quantum chemistry-based calculations of NMR displacement theory with a Bayesian approach that assigns accurate probabilities to potential structures and incorporates objective model selection for peak picking and noise reduction.^475^

One of the most crucial applications of AI in drug discovery is the prediction of macromolecular targets, their associated biological activity, and potential toxicity. Accurate predictions in these areas offer direct insights into the most promising regions within chemical space for drug discovery. Numerous methods rely on classical cheminformatics and computer-aided drug discovery tools to predict the biological activity of drugs. However, owing to the unique chemical structure and physicochemical properties of drugs, successful applications often require additional pretreatment steps or consideration of unknown chemical disparities between training data from natural products and synthetic compounds.^476^ Structure-guided approaches utilize spatial information about protein targets obtained from experimentally determined structures or through deep learning-based modeling methods to predict the binding patterns of compounds. Subsequently, strategies such as molecular dynamics simulations can be employed to calculate potential binding modes by docking protein dynamics. Notably, FEP methods have recently gained significant popularity in both academic and industrial drug discovery projects.^477^

The discovery of new data-driven AI relies heavily on the long-term preservation and maintenance of databases. Although high-quality data are essential for AI, obtaining consistent financial support for maintaining databases can be challenging. Therefore, international and national funding agencies must prioritize continued support for database maintenance and interoperability to facilitate future advancements in AI.

A typical drug design project based on protein structure commences with the protein sequence and proceeds to construct a 3D structure via structural biology or structural prediction techniques. The binding pocket is subsequently identified, encompassing both normal and allosteric sites, culminating in the discovery of active modulators through virtual screening or de novo design.

The concept of computational drug design, which is grounded in protein sequence information, has significantly advanced with the introduction of TransformerCPI2.0 by Mingyue Zheng et al., as depicted in Fig. 13b. This transformative approach enables TransformerCPI2.0 to effectively interpret binding knowledge, identify novel targets for challenging drug candidates, and discover potential targets for existing drugs through reverse application of the concept. Consequently, it serves as a valuable complement to structure-guided drug design, particularly for proteins lacking a well-defined 3D structure.^478^

Currently, AI has demonstrated significant potential in the field of drug discovery; however, it continues to face multiple challenges and limitations. From a technical perspective, issues such as insufficient model accuracy and reliability—particularly in handling flexible targets and predicting affinity—persist. The constraints of generative AI (wherein molecules generated by AI may be unsynthesizable, possess unknown toxicity, or exhibit poor drug-like properties) further hinder progress in this domain. Additionally, models such as deep neural networks are often regarded as black boxes, making it difficult to interpret their decision-making logic. This lack of transparency limits scientists’ trust in these models and hampers optimization efforts. Moreover, there are ongoing concerns regarding data quality and diversity, alongside issues related to data privacy and compliance. Preclinical and clinical data tend to be fragmented with varying quality standards. Pharmaceutical companies’ confidentiality policies (such as nondisclosure of clinical outcomes) result in a lack of feedback mechanisms for optimizing AI models.

In the future, developing multimodal fusion models that integrate protein structures and gene expression data to enhance interpretability is essential. Additionally, exploring quantum computing for accelerating molecular simulations is crucial. The establishment of industry alliances or public databases should be promoted to address the issue of data silos. By combining biology, chemistry, and AI technologies, researchers can create a closed-loop system of AI + experimentation that reduces trial-and-error costs. These initiatives are expected to effectively address the aforementioned challenges. Therefore, despite facing multiple obstacles, AI continues to be regarded as a disruptive tool in drug discovery. Moving forward, it is necessary to achieve gradual breakthroughs through technological iteration, policy support, and industry collaboration—transforming AI from an auxiliary tool into an innovation engine.

Issues related to data privacy, algorithmic bias, social equity, and the delineation of responsibility extend beyond mere technical considerations—they pertain to fairness, privacy, and accountability. In confronting these challenges, researchers require not only technological breakthroughs but also deep reflections on ethical and societal dimensions. While employing these technologies, it is imperative that we prioritize data privacy protection and ensure the transparency, interpretability, and fairness of AI models. Furthermore, an independent ethical review mechanism should be integrated at every stage of drug discovery. Researchers must strive to find a balance between efficiency and responsibility to ensure that the application of technology genuinely serves the welfare of all humanity rather than becoming a source of new inequalities and risks.

### Nanotechnology

Nanotechnology has played a pivotal role in various aspects of the battle against viruses, facilitated by the successful development of two highly effective messenger RNA vaccines based on nanotechnology.^479^ The utilization of nanoparticles with neutralizing antibodies on their surface and nanosheets as novel drugs against SARS-CoV-2 infection not only provides valuable insights and design strategies for combating future variants but also holds great potential in driving innovations toward nanotechnology-based approaches to address other global infectious diseases.^480^

Nanozymes are a type of nanomaterial possessing inherent enzyme-like properties, exhibiting immense potential in diverse biomedical applications, such as disease diagnosis and antiviral therapeutics. Single-atom nanozymes (SANs) refer to simulated enzymes comprising isolated single metal atoms dispersed on various substrates, thereby maximizing the atomic utilization efficiency and active site density. Guohui Nie et al. demonstrated that Ag-TiO_2_ SAN has remarkable anti-SARS-CoV-2 activity. Theoretical calculations and experimental evidence suggest that Ag atoms within SAN strongly interact with cysteine and asparagine, which are the predominant amino acids present on the surface of the S1 protein. In vivo studies revealed that SAN effectively enhances virus phagocytosis by macrophages. Moreover, the intrinsic peroxidase-like activity of Ag-TiO_2_ SAN results in potent antiviral effects within lysosomes. Notably, in a virus elimination model, Ag-TiO_2_ SAN significantly eradicates pseudoviruses while safeguarding host cells against infection.^481^

Nanotechnology has significantly enhanced the efficacy and safety of antiviral drugs through precise delivery, targeted therapy, and direct antiviral action. The potential of mRNA vaccines, metal nanoparticles, and intelligent nanosystems has been demonstrated in clinical settings and during epidemics. In the future, it is essential to integrate AI in the design of novel nanomaterials to optimize delivery efficiency while overcoming challenges related to biocompatibility and large-scale production. This approach will be crucial in addressing threats posed by viral mutations and emerging infectious diseases.

### Nucleic acid drugs

Nucleic acid drugs encompass a diverse range of RNAs or DNAs with distinct functionalities, primarily operating at the genetic level. This category comprises aptamers, antigen agents, ribozymes, antisense nucleic acids, and RNA interference agents.^482^ Owing to their precise targeting of pathogenic genes, these therapeutics exhibit specific targets and mechanisms of action.

Aptamers, as single-stranded (ss) RNA or DNA oligonucleotides, can adopt unique 3D structures through structural recognition and exhibit high binding affinity toward diverse targets.^483^ The aptamer blocking strategy presents a viable solution to mitigate the risk of antibody-dependent enhancement and unfavorable size for intranasal delivery of SARS-CoV-2 neutralizing antibodies. Chaoyong Yang et al. demonstrated that the aptamer has high affinity for the receptor-binding domain of the S protein (*K*_d_ = 0.13 nM) and effectively inhibits viruses with an IC_50_ value of 0.42 nM.^484^ Aptamers have also been reported in HIV-1, exhibiting selective binding to the CA lattice and thereby inhibiting HIV-1 replication.^485^

## Conclusion and perspectives

At present, effective treatment strategies that are inexpensive and convenient for addressing the virus are still needed, including strategies that can address drug resistance caused by new mutations. We summarize the advancements and future trends of antiviral drug discovery, hoping to provide some help for drug development. These strategies include technologies that target novel sites and exploit solvent-exposed regions, substrate structures, covalent binding strategies, and multivalent ligands. Furthermore, emerging approaches such as virtual screening and targeted protein/RNA degradation have been harnessed to curtail the emergence of drug resistance.

The design of broad-spectrum antiviral drugs is a complex and intricate process, encompassing target identification, candidate drug screening and design, validation of drug activity, optimization of drug performance, and clinical trials. A commonly employed strategy involves the screening of therapeutic compounds with established safety profiles. Furthermore, drugs can be designed to target key host factors involved in the viral replication cycle to exert a wide-ranging effect and mitigate the emergence of drug resistance. However, owing to the vast diversity and variability of viruses, developing a drug that effectively targets multiple viruses simultaneously poses significant challenges. The complexity of drug discovery, combined with the integration of emerging technologies, has positioned interdisciplinary collaboration as a core driving force for innovation. This collaborative approach has evolved from an optional model to an essential pathway in the field of drug discovery.

The process of drug development involves not only technical challenges but also the need for pharmaceutical companies to engage in early communication with regulatory authorities. By understanding regulatory requirements and approval standards from the outset, these companies can better plan their research directions and strategies, thereby avoiding unnecessary adjustments and delays later. For instance, during the drug design phase, companies can discuss aspects such as the mechanism of action and potential risks with regulatory bodies to ensure that their research aligns with compliance expectations. Furthermore, supporting international multicenter clinical trials allows for simultaneous research across multiple countries and regions. This approach not only accelerates data collection and analysis but also enhances the global recognition and marketability of new drugs.

The application of computer technology, modern synthesis techniques, biotechnology, and rapid advancements in disciplines such as molecular biology, genetics, and immunology have significantly contributed to the theory and practice of medicinal chemistry. The interconnection and integration among these disciplines have further enhanced its scientific foundation and injected renewed vigor into this field. Moreover, emerging technologies such as targeted protein/RNA degradation methods (e.g., LYTAC, ATTEC), as well as novel approaches targeting cell substructures (exosomes, lysosomes), nuclear export pathways, the cell membrane, and RNA molecules, offer promising opportunities for advancing medicinal chemistry. In the coming decade, the convergence of these emerging technologies is poised to usher in a new era of drug discovery characterized by efficiency, precision, and personalization. While challenges remain, collaborative innovation among these technologies has the potential to reshape the pharmaceutical industry and provide groundbreaking therapies for viral diseases, significantly enhancing human health.

In particular, the increasing utilization of advanced information technology in the field of biology has led to a vast array of relevant data and tools, enabling AI pharmaceuticals to explore diverse development avenues and wider application domains. While the exact extent of AI’s impact on the pharmaceutical industry remains uncertain, it is undeniable that AI pharmaceuticals have been steadily advancing for over a decade. The pursuit of life and health by humans is perpetual, and with AI being one of the most significant technological advancements in the forthcoming decades, it will undoubtedly expedite this process—a prospect we approach with great enthusiasm and confidence.

Unlike conventional drug discovery approaches, biomolecular condensate-based drug discovery represents a comprehensive and innovative pathway that integrates multiple advantages, including target identification and phenotypic screening. This approach not only offers an alternative interpretation of the pathogenic mechanisms underlying specific targets but also expands the scope of “druggable” target screening by introducing novel biological phenotypes. Furthermore, in cases where molecular disease pathology remains elusive, condensate-based phenotypic screening strategies can provide promising therapeutic options. Given the nascent stage of this field, research across all facets is still in its infancy. As this field continues to evolve, we anticipate gaining deeper insights into the dynamic interaction network governing biomolecular condensates and gradually transitioning most biomolecular condensates from being primarily phenotype oriented to being target focused.
